# One-Carbon Metabolism in Prostate Cancer: The Role of Androgen Signaling

**DOI:** 10.3390/ijms17081208

**Published:** 2016-07-27

**Authors:** Joshua M. Corbin, Maria J. Ruiz-Echevarría

**Affiliations:** 1 Department of Pathology, Oklahoma University Health Sciences Center, Oklahoma City, OK 73104, USA; Joshua-Corbin@ouhsc.edu; 2 Department of Pathology, Oklahoma University Health Sciences Center and Stephenson Cancer Center, Oklahoma City, OK 73104, USA

**Keywords:** one-carbon metabolism, androgen receptor, epigenetics, methylation, polyamine metabolism, transsufluration

## Abstract

Cancer cell metabolism differs significantly from the metabolism of non-transformed cells. This altered metabolic reprogramming mediates changes in the uptake and use of nutrients that permit high rates of proliferation, growth, and survival. The androgen receptor (AR) plays an essential role in the establishment and progression of prostate cancer (PCa), and in the metabolic adaptation that takes place during this progression. In its role as a transcription factor, the AR directly affects the expression of several effectors and regulators of essential catabolic and biosynthetic pathways. Indirectly, as a modulator of the one-carbon metabolism, the AR can affect epigenetic processes, DNA metabolism, and redox balance, all of which are important factors in tumorigenesis. In this review, we focus on the role of AR-signaling on one-carbon metabolism in tumorigenesis. Clinical implications of one-carbon metabolism and AR-targeted therapies for PCa are discussed in this context.

## 1. Introduction

Prostate cancer (PCa) is the most frequently diagnosed non-skin cancer and the fifth leading cause of cancer death in men worldwide [1]. Clinically, PCa is a heterogeneous disease, ranging from an indolent disease, requiring no treatment, to highly aggressive PCa that develops into metastatic disease. Despite this heterogeneity, prostate tumor growth is, almost always, dependent upon the androgen receptor (AR) pathway [2,3,4], explaining the efficacy of androgen deprivation therapies (ADT) or anti-androgens for the treatment of hormone-naïve PCa [5,6]. However, most patients relapse following ADT and the disease progresses to castration-resistant prostate cancer (CRPC), which is lethal [7,8,9]. Central to the development of CRPC is the reactivation/adaptation of AR signaling to function under low androgen levels. Therefore, the AR and the processes downstream of the AR remain as targets for therapeutic intervention throughout the different stages of the disease. Recent results indicate that the AR drives a distinct transcriptional program in CRPC, and that changes in AR activity are critical to drive disease progression [10,11]. Efforts to identify clinically relevant, AR-modulated, transcriptional networks have established a link between the AR and cellular metabolism, consistent with the changes in metabolism that occur with disease progression [12,13]. Recent data indicate that expression of the constitutively active AR-V7 variant in CRPC has novel metabolic functions that may be specifically targeted [14].

In PCa, the one-carbon metabolism pathway is modulated by the AR. This pathway is comprised of several connected pathways that promote the folate-mediated transfer of one-carbon units necessary for essential cellular processes including DNA synthesis and repair and the maintenance of redox status. Because one-carbon metabolism is also the major source of methyl groups, as a modulator of this pathway, the AR also plays critical roles in histone and DNA methylation and in epigenetic mechanisms that are known to be relevant in oncogenesis [15,16,17]. Studies in PCa cell lines demonstrate AR-regulation of one-carbon metabolism enzymes, and altered cellular methylation potential in response to androgens [18,19,20,21]. In PCa clinical samples, accumulation of sarcosine, a methylated metabolite of the one-carbon pathway, correlates with disease progression [20]. Changes in several other metabolites also correlate with PCa risk [22]. These findings illustrate the role of the AR in PCa tumorigenesis by controlling metabolism, and the value of integrating metabolomic profiling and gene expression analysis for the identification of new biomarkers and therapeutic targets.

In this review, we will focus on the role of the AR on one-carbon metabolism and the implications for disease progression. The first two sections focus on the relevance of one-carbon metabolism and its link to cancer. The third section outlines how AR-signaling modulates the expression and activity of enzymes involved in one-carbon metabolism, and how it affects methylation-mediated epigenetic processes in PCa. The final section discusses targeting one-carbon metabolism in PCa, and the potential effects of current AR-targeting therapeutic modalities on one-carbon metabolism.

## 2. The One-Carbon Metabolism Network

**Figure 1:**
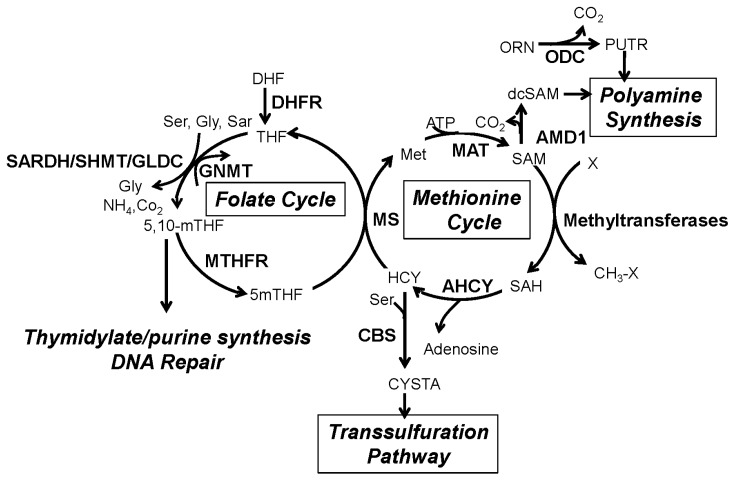
One-carbon metabolism and associated pathways. One-carbon metabolism involves the transfer of methyl groups to various substrates and cofactors within the folate and methionine cycles, and the polyamine biosynthetic and transsulfuration pathways. Methyl groups are utilized in the synthesis of nucleotides, and polyamines, as well as, DNA and protein methylation reactions. Enzymes are depicted in bold, while metabolites/substrates/cofactors are in regular font. Enzyme abbreviations are as follows: DHFR: Dihydrofolate reductase; SARDH: Sarcosine Dehydrogenase; SHMT: Serine hydroxymethyltransferase; GLDC: Glycine decarboxylase; GNMT: Glycine-*N*-methyltransferase; MTHFR: Methylene tetrahydrofolate reductase; MS: Methionine synthase; MAT: Methionine adenosyltransferase; AMD1: Adenosylmethionine decarboxylase; ODC: Ornithine decarboxylase; AHCY: S-adenosylhomocysteine hydrolase; CBS: Cystathionine beta-synthase.

One-carbon metabolism involves a complex network with two central cycles: (1) the folate cycle; and (2) the methionine cycle (Figure 1). In the folate cycle, tetrahydrofolate (THF) acts as a carbon carrier donor for the synthesis of purines and thymidilates, which are vital for DNA synthesis and repair. The transfer of methyl groups from 5-methylTHF to homocysteine to form methionine links the two cycles. Methionine is then converted to *S*-adenosyl-methionine (SAM), the universal methyl donor for protein and DNA methyltransferase reactions. By donating a methyl group, SAM is converted to *S*-adenosyl-homocysteine (SAH), and subsequently to homocysteine to close the cycle [17,23,24,25]. In addition to being recycled back to methionine, homocysteine can also be shunted to the transsulfuration pathway where it is converted into cystathionine, a precursor of glutathione, an important cofactor in oxidation/reduction (redox) reactions that regulate the cellular redox state. SAM can also contribute to the synthesis of polyamines, which are small organic cations that regulate multiple biological processes, including, translation and proliferation, linking the methionine cycle with polyamine synthesis [26,27]. Since one-carbon metabolism regulates essential processes including DNA synthesis and repair, epigenetic methylation reactions, redox homeostasis, and protein synthesis, the balanced flux through these four pathways (folate cycle, methionine cycle, transsulfuration pathway, polyamine synthesis) is essential for cellular homeostasis. In fact, disruptions in that balance contribute to the pathogenesis of many diseases, including cancer [28].

**Figure 2:**
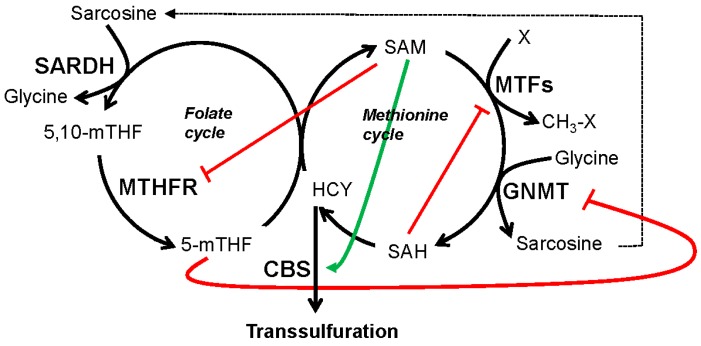
Regulation of the one-carbon metabolism maintains a balanced flux between the folate and methionine cycles and associated pathways. Metabolites produced within the folate and methionine pathways regulate the activity of the enzymes within the one-carbon metabolism network to maintain the balance of methyl groups and metabolites within the folate and methionine cycles and associated pathways and to allow for changes in response to cellular demands or growth conditions. See text for details. Enzymes are in bold, and substrates/cofactors are depicted in regular font. Black arrows indicate the directionality of reactions, red lines indicate inhibition, and the green arrow indicates activation. Enzyme abbreviations are as follows: SARDH: Sarcosine dehydrogenase; MTHFR: Methylene tetrahydrofolate reductase; CBS: Cystathionine beta-synthase; MTFs: Methyltransferases; GNMT: Glycine-*N*-methyltransferase.

Balance within the one-carbon metabolism network is maintained in part by interactions involving substrates and enzymes from these pathways (Figure 2). SAM inhibits methylene-tetrahydrofolate reductase (MTHFR), the enzyme that catalyzes formation of 5-methylTHF, a necessary cofactor to regenerate methionine and, ultimately, SAM levels [17]. 5-methylTHF is an inhibitor of glycine *N*-methyltransferase (GNMT), the enzyme that catalyzes formation of sarcosine from glycine, which eventually donates methyl groups back to the THF in a reaction catalyzed by sarcosine dehydrogenase (SARDH) [29,30]. SAM also stimulates cystathionine beta-synthase (CBS), the enzyme that shuttles homocysteine into the transsulfuration pathway [31,32]. Additionally, folate regulates enzymes involved in polyamine metabolism [33,34]. These interactions maintain an exquisite balance between one-carbon metabolism and its associated pathways to maintain cellular homeostasis.

## 3. One-Carbon Metabolism in Cancer

Cancer creates a demand and dependency on one-carbon metabolism. Proliferation of tumor cells not only requires increased DNA synthesis, but can also result in increased levels of reactive radical oxygen species (ROS), which are cytotoxic unless neutralized [35,36]. Methyl group availability for methyltransferases that modulate gene expression via epigenetic mechanisms is influenced by flux within the folate cycle and methionine cycles [15,16]. In addition, synthesis of polyamines, which have been suggested to have oncogenic functions through regulating protein synthesis and proliferation [37,38], is SAM-dependent.

Several enzymes within the folate cycle are potentially oncogenic and are dysregulated in cancer. Serine hydroxymethyltransferase (SHMT) and glycine decarboxylase (GLDC) donate methyl groups to the folate pathway in sequential steps via the catabolism of serine and glycine, respectively [15]. SHMT, in concert with GLDC, drives tumorigenesis possibly by fueling the folate cycle and driving proliferation [39]. Thymidylate synthase (TS), another enzyme involved in the folate cycle, catalyzes the methylation of deoxyuracil-monophosphate to deoxythymidine-monophosphate, in a 5,10-methylene-THF-dependent reaction that is necessary for DNA synthesis and repair. The overexpression of TS is sufficient to induce a tumorigenic phenotype in NIH3T3 cells in vivo, and elevated TS expression correlates with a poor prognosis in multiple cancer types [40,41,42,43,44,45,46]. Furthermore, the TS inhibitor, 5-fluorouracil (5-FU), is used in the treatment of multiple cancers, especially colon cancer [47].

Paradoxically, although folate is necessary for cancer cell proliferation, multiple studies have reported a positive correlation between folate deficiency and disease risk for multiple cancers, especially breast and colon cancers [48,49,50,51]. Additionally, higher folate intake reduces the increased breast cancer risk associated with elevated alcohol consumption; this relationship may be due in part to the antagonistic effect of alcohol on folate absorption, metabolism and transport [51]. Aberrant uracil incorporation and chromosomal breaks can both be induced by folate deficiency, thus providing a potential mechanism by which folate deficiency can contribute to tumorigenesis [52,53]. Additionally, the MTHFR C677T polymorphism may be associated with increased breast cancer risk [49,54,55,56,57,58]. The C677T polymorphism reduces MTHFR activity, thus lowering 5-methyl-THF levels and decreasing methionine regeneration [59]. Not only can folate deficiency contribute to mutations during replication [53,60], but folate deficiency or MTHFR polymorphisms may also decrease methionine regeneration and SAM levels, thereby, reducing the ability of the cell to maintain DNA and histone methylation. Importantly, cancer cells often exhibit global DNA hypomethylation, a phenotype that may be linked to genomic instability [52,61,62].

In contrast, folate depletion blocked tumor progression in vivo and induced genetic instability in cells in vitro, in the Transgenic Adenocarcinoma of the Mouse Prostate (TRAMP) model for PCa [63,64]. Further, folate supplementation has been shown to drive tumor growth in some mouse and rat cancer models [65,66,67]. However, the timing of folate supplementation in disease progression is likely critical, as studies indicate that folate may be both protective against neoplastic lesion formation and a promoter of growth within established lesions [67,68,69]. These studies highlight a widely supported “double-edged sword” hypothesis for the role of the folate cycle in cancer: Folate depletion may contribute to initial transformation by inducing global DNA hypomethylation and subsequent genomic instability, while higher folate levels may promote the growth of transformed cells by enabling an increased rate of DNA synthesis [68,69].

Even in the presence of global DNA hypomethylation, many cancer cells contain gene specific hypermethylation, a silencing mechanism. The tumor suppressor Rb was the first gene found to be silenced by DNA hypermethylation during tumorigenesis [70]. Since then, numerous tumor suppressor genes have found to be silenced by DNA hypermethylation in cancer. Unlike DNA mutations, epigenetic aberrations—including DNA methylation—can be reversed by inhibiting the enzymes responsible for the epigenetic marks. This is one reason why targeting epigenetic enzymes has gained traction in cancer therapy [71].

Histone methylation is a SAM-dependent epigenetic process. Several methyl histone marks are dysregulated in many cancer types, and depending on the target residue, these methylated histones can contribute to gene activation or repression [72,73,74,75,76,77]. The enhancer of zeste homolog 2 (EZH2), DOT1L and mixed-lineage leukemia (MLL) methyltransferases are among the histone methyltransferases (HMTs) found to play important roles in driving a tumorigenic epigenome, which is similar to that of stem cells [78,79,80,81,82,83,84,85,86] HMTs use SAM as a methyl donor, and many HMTs are inhibited by SAH (Figure 2), a byproduct of methyltransferase reactions; therefore, one-carbon metabolism flux has a profound impact on the activity of these enzymes [87,88,89].

SAM not only serves as a cofactor for methyltransferases, but it is also shunted from one-carbon metabolism and utilized in polyamine synthesis. Polyamines have been implicated in cancer, and their oncogenic function may be linked to their roles in protein synthesis and cell cycle regulation [27,37,90]. Ornithine decarboxylase (ODC) catalyzes the formation of putrescine from ornithine, a rate-limiting step in the polyamine biosynthetic pathway. ODC is a MYC-regulated oncogene that is critical for cell cycle progression, in part by promoting MYC-induced p21 degradation [91,92].

Another shunt from the methionine cycle is the transsulfuration pathway, which is important for cellular redox homeostasis. The high intracellular oxygen levels required for aerobic respiration create an environment that produces highly reactive ROS. While physiological levels of ROS are essential for cell survival, an excess of ROS can have a wide range of detrimental effects, including DNA and protein damage. To prevent damage, the cell tightly regulates a series of antioxidant systems to restore redox homeostasis. One of the major antioxidants made within cells is glutathione, which is a product of the transsulfuration shunt of one-carbon metabolism. Reduced glutathione acts a cofactor for redox and conjugation reactions catalyzed by glutathione peroxidases and glutathione transferases to reduce hydrogen peroxide, a reactive product of initial superoxide neutralization, and neutralize toxins and carcinogens. Interestingly, in multiple cancers, glutathione peroxidases and glutathione transferases are silenced by DNA hypermethylation suggesting that the reduced activity of the enzymes drives tumorigenesis, likely through increased DNA damage [93,94,95,96,97,98]. However, the overexpression of glutathione peroxidases and glutathione transferases, along with elevated levels of reduced glutathione, has been observed to correlate with therapy resistance in multiple cancers [99,100,101]. This evidence suggests that the glutathione-dependent reduction and neutralization reactions may have complex pro-tumor and anti-tumor effects by improving survival and reducing DNA damage.

Interestingly, elevated homocysteine may promote oxidative stress by inhibiting the expression and activity of glutathione peroxidases. Elevated plasma homocysteine levels, a condition that may also be associated with folate deficiency, is often seen in the setting of malignancy [48,102,103,104,105,106,107]. In addition to being a metabolite that is utilized in glutathione synthesis, homocysteine regulates the activity of enzymes that use glutathione as a cofactor. By controlling glutathione synthesis and utilization, changes in one-carbon metabolism flux can have a profound impact on redox metabolism, and therefore, potentially tumorigenesis and cancer progression.

Taken together, alterations in one-carbon metabolism may contribute to tumorigenesis by fueling DNA synthesis, changing the DNA and histone methylomes, promoting protein translation, driving cell cycle progression and modulating redox balance. These changes can in turn promote sustained proliferation, induce tumorigenic gene expression changes, contribute to genomic instability, and promote survival—all important processes in tumorigenesis and cancer progression.

## 4. Androgen Signaling Modulates One-Carbon Metabolism and Epigenetics

In the prostate, androgens and the AR regulate the activity/expression of several enzymes involved in the one-carbon metabolism pathways, specifically enzymes involved in SAM homeostasis (GNMT and SARDH) and the entry into the transsulfuration (CBS) and polyamine synthesis (ODC) pathways (Figure 1 and Table 1). This suggests that the changes in the AR activity that occur during PCa progression may have profound effects on global one-carbon metabolism and the epigenetics of this disease. In this section, we review the role of androgens/AR signaling in these checkpoints of the one-carbon metabolism network, with an emphasis on the effect on gene expression and focusing on the best characterized genes. Based on the impact of the one-carbon metabolism in epigenetics, we will also discuss the effect of androgen signaling on the activity/expression of methyltransferases and epigenetic processes in PCa

## 5. Androgen Signaling Regulates the Expression of Enzymes Involved in the One-Carbon Metabolism Network

The AR is a nuclear receptor that is essential for prostate differentiation and homeostasis and for PCa initiation and progression. Binding of androgen, its major ligand, triggers a conformational change that promotes AR homodimerization and translocation to the nucleus, where it binds to the regulatory regions of its target genes, affecting their transcription [108]. Studies directed to identify AR transcriptional networks in different models of PCa have demonstrated an involvement of the AR in global metabolism by directly targeting enzymes involved in several metabolic processes [12,13,109,110]. Below we focus on several specific AR targets involved in one-carbon metabolism and their role in PCa.

### 5.1. GNMT, SARDH and Sarcosine Metabolism

GNMT catalyzes the transfer of a methyl group from SAM to glycine to form SAH and sarcosine. The reverse reaction involves the oxidative demethylation of sarcosine into glycine, and it is catalyzed by mitochondrial SARDH or peroxisomal PIPOX [19,111]. It has been proposed that the “sarcosine cycle” and GNMT in particular regulate the SAM:SAH ratio, and therefore the methylation potential of the cell [111]. Methyltransferases are inhibited by SAH [87], GNMT is allosterically inhibited by 5-methylTHF [30], and SAM inhibits MTHFR and therefore formation of 5-methylTHF [111]. When SAM levels are low, this regulatory loop promotes release of the inhibition of MTHFR, resulting in de novo synthesis of 5-methylTHF and therefore ensuring inhibition of GNMT so that SAM will be saved for physiologically essential methylation reactions. High levels of SAM block formation of 5-methylTHF, releasing the inhibition of GNMT, which will convert excess SAM into sarcosine [111]. Because of the relevance of GNMT and the sarcosine cycle in methylation, changes in their expression or activity can have profound effects in essential cellular processes. The AR and the TMPRSS2-ERG fusion product (present in over 50% of localized PCa and whose expression is controlled by the AR) are known to coordinately regulate GNMT and SARDH expression [20,21]. Therefore, as expression/activity of these transcription factors changes with disease progression, so does the methylation potential of the cell. In fact, the role of GNMT and SARDH in PCa has gained recent interest, as both are dysregulated during tumorigenesis and control the metabolism of sarcosine. Sarcosine is a metabolite that increases during PCa progression to metastasis, and has been proposed as a potential non-invasive urine biomarker [20]. Using PCa cell lines, Sreekumar et al. [20] demonstrated that the enzymes involved in sarcosine metabolism act as regulators of cell invasion and are therefore potential therapeutic targets for prostate cancer. The addition of sarcosine or knockdown of SARDH in benign prostate epithelial cells enhanced their invasiveness. Recently, we demonstrated that sarcosine metabolism, not merely its concentration, and thus one-carbon availability, is responsible for the changes in invasion observed in PCa cells [18].

While controversy remains regarding whether the levels of GNMT in clinical PCa samples are downregulated [112] or upregulated [113], it is clear that dysregulation of GNMT may reflect changes in AR activity and ERG fusion status during PCa establishment and progression. Metabolomic analyses indicate that androgen supplementation results in elevated amino acid metabolism and increased methylation activity in PCa cells [114,115]. Interestingly, in breast cancer, the expression of sarcosine-related enzymes has been shown to vary according to cancer subtype [115]. A parallel with GNMT could be established with studies conducted on Nicotinamide *N*-methyltransferase (NNMT) [116]. NNMT, which catalyzes the transfer of a methyl group from SAM to nicotinamide to generate 1-methylnicotinamide (1-MNA) and SAH, and its products, are overexpressed in several aggressive cancer cell lines (e.g., ovarian, lung, and kidney) and in clinical samples [117]. Similar to sarcosine, 1-MNA does not have a known physiological role, but has been proposed to act as a sink for methyl groups, reducing the SAM:SAH ratio and the methylation potential of the cell [116]. The authors demonstrated that NNMT overexpression led to decreased methylation of proteins including histones, and associated changes in gene expression. It is possible that when GNMT is overexpressed and SARDH is underexpressed or its activity is decreased (as previously postulated for aggressive behavior in PCa; [20]), overproduction of sarcosine can exert a similar “methyl sink” effect. In this regard, we have previously demonstrated that the transmembrane protein with epidermal growth factor and two follistatin domains 2 (TMEFF2) is a tumor suppressor that cooperates with SARDH to modulate one-carbon metabolism in PCa cells [18,118] suggesting that additional factors may play a role in the activity of these enzymes. Metabolic changes in a TMEFF2 transgenic mouse model support this conclusion [119].

### 5.2. CBS and the Transsulfuration Pathway

As discussed above, homocysteine can enter the transsulfuration pathway in a reaction that involves condensation with serine, resulting in cystathionine. In mammals, this first and committed step of the pathway is catalyzed by CBS. The second step, the hydrolysis of cystathionine to cysteine, is catalyzed by the enzyme γ-cystathionase [120]. Cysteine is a limiting factor for glutathionine synthesis, but can also be catabolized via other routes, including a non-oxidative route that produces hydrogen sulfide (H2S). H2S plays a role in the regulation of many physiological processes, such as the cellular stress response, inflammation and energy metabolism [121,122,123,124], and it modulates AR activity [125]. Based on its roles in homocysteine homeostasis and H2S and glutathione generation, altered CBS activity/expression contributes to numerous diseases, including cancer [126,127,128].

The activity of CBS is stimulated by SAM binding [31,32,129], so that homocysteine metabolism can be directed towards remethylation when methionine/SAM levels are low, and towards the transsulfuration pathway when SAM levels are high. Studies using LNCaP, an androgen-dependent prostate cancer cell line, suggest that CBS expression may be downregulated by androgens via a currently unknown posttranscriptional mechanism and that this effect is accompanied by a decrease in glutathionine levels [130,131]. Reduced levels of CBS have also been reported in the metastatic PCa cell line PC3. However, this cell line does not express the AR, and the low levels of CBS did not seem to correlate with the cancer phenotype [132]. In addition, lower levels of plasma cysteine have been observed as a result of prostate tumor progression in mouse xenografts [133]. The above findings that suggest an impaired flux through the transsulfuration pathway in PCa are not supported by clinical metabolomic data. In a study analyzing metabolite levels in serum of patients who developed recurrent disease after primary treatment vs. patients that remained recurrence-free, the levels of homocysteine and cystathionine were significantly higher in the recurrent group than in the recurrence-free group [134]. Increased levels of homocysteine and methylated metabolites, with concomitant decrease in SAM, were observed in androgen-responsive PCa cells when compared with PCa cells that were non-responsive to androgens [114]. The levels of H2S are also significantly higher in patients with localized PCa than in patients with benign prostatic hyperplasia or healthy individuals [135]. These results suggest an androgen-mediated increase of methylation activity and an increased flux through the transsulfuration pathway in PCa and with the progression to aggressive disease. Reconciling these seemingly opposite results requires determining the role of transsulfuration metabolites in cancer, analyzing differences in methylation potential across individuals, and establishing the role of SAM and androgen signaling changes with disease progression in the one-carbon metabolism and transsulfuration pathways.

As we discussed earlier, the AR plays a role in regulation of GNMT. Thus, in modulating GNMT activity, the AR indirectly control homocysteine levels and the SAM:SAH ratio, critical to methylation reactions and to the level of CBS. H2S inhibits the activity of the AR [125] providing a feedback loop by which excess cysteine and, therefore H2S, modulates AR activity and the methylation and transsulfuration pathways. In hepatic and lymphocytic cells, androgens have been demonstrated to regulate expression of glutathione *S*-transferase Pi (GSTP), an enzyme with a role in detoxification, by catalyzing the conjugation of many compounds to reduced glutathione [136,137,138]. Consequently, the AR can play a role in detoxification not only by regulating CBS levels, and thus glutathione, but also by regulating/modulating the activity of enzymes that act downstream of glutathione. Changes in ROS are known to have a role in the etiology and progression of PCa [139].

### 5.3. ODC, SAM and Polyamine Synthesis

The relevance of polyamines to cellular physiology is illustrated by the fact that knockout of several enzymes of the pathway are embryonic lethal in the mouse [140] and dysregulation of polyamine metabolism leads to disease [141]. Increased levels of polyamine synthesis and ODC levels have been associated with cancer and other hyperproliferatives diseases [37,91,142,143,144]. ODC catalyzes the initial and rate limiting step in the biosynthesis of polyamines, a conversion of ornithine to putrescine. Sequential reactions catalyzed by spermidine and spermine synthase convert putrescine into spermidine and spermine, respectively. These reactions require dcSAM, which is obtained from the decarboxylation of SAM in a reaction catalyzed by SAM-decarboxylase (*AMD1*; Figure 1).

The prostate has exceptionally high levels of polyamines, which are synthesized in the epithelium for normal growth and for secretion into the seminal fluid [26,38,145,146,147,148]. The high level is due, in part, to the high expression of ODC and AMD1 [145,146,149,150,151]. Both enzymes, together with spermidine synthase, are induced transcriptionally by androgens/AR signaling in the prostate in a coordinated way [152,153,154,155,156]. Moreover, ODC is higher in PCa than in benign tissue, tissue from patients with benign prostate hyperplasia (BPH), or tissue from normal volunteers [149,157], indicating that changes that occur to the AR during PCa progression affect enzyme levels and polyamine synthesis. Providing further evidence for this notion, androgen-blocking therapies, inhibit production of spermine and spermidine [158,159].

The high polyamine requirements observed in the prostate, which are increased in PCa, sensitize the cells to folate levels [160]. Blocking polyamine synthesis by inhibiting AMD1 increases SAM levels and reduces the sensitivity to low levels of folate [160]. Interestingly, mild folate deficiency does not negatively impact polyamine levels, but does affect DNA methylation and cell growth, suggesting that maintaining polyamine pools is favored over maintaining SAM pools [63,160]. Due to the high demand for polyamines in the prostate and in PCa, changes in AR-mediated polyamine biosynthesis enzyme levels can create an imbalance in SAM levels and nucleotide pools, having profound effects on DNA damage, DNA methylation, and other epigenetic changes, leading to tumorigenesis and/or playing a role in disease progression (see below).

## 6. The Role of Androgen Signaling on Methyltransferases and the Epigenetics of PCa

DNA and histone methylation are important epigenetic mechanisms that contribute to initiation and progression of PCa [75,161,162,163,164]. Based on the link between these epigenetic mechanisms and one-carbon metabolism, in this section we briefly review the role of methylation in PCa and discuss how the AR modulates the epigenetics of PCa, indirectly controlling one-carbon metabolism and directly affecting the expression and activity of methyltransferases.

### 6.1. DNA and Histone Methyltransferases in PCa

In PCa, changes in DNA methylation are detected before the cancer becomes invasive and are maintained throughout disease progression [165,166]. These observations underscore the relevance of epigenetic mechanisms to PCa and suggest that epigenetic changes are early events that may even be responsible for PCa tumor initiation. The best-characterized epigenetic alteration in PCa is gene-specific DNA hypermethylation [167,168]. Aberrant hypermethylation of numerous genes including cell cycle control genes, detoxification and genes involved in apoptosis and DNA repair [166,167,168,169,170,171,172,173,174,175,176] and the AR itself [172,177,178,179,180] has been described. Correspondingly, expression and activity of DNA methyltransferase 1 (DNMT1), the methyltransferase that is primarily responsible for maintaining the DNA methylation pattern, is higher in localized, metastatic, and hormone-resistant PCa samples than in benign prostate hyperplasia (BPH) or normal tissue. DNMT1 level can predict disease recurrence after prostatectomy [167,168,175,180,181,182,183]. Changes in the level of DNMT1 with disease progression have also been reported in studies using the TRAMP mouse model [184]. Using this model, it was also demonstrated that inhibition of DNMT1 by 5-azacitidine treatment prevented tumorigenesis [185], underscoring the relevance of DNMT1 and hypermethylation to the establishment and progression of PCa. Expression of other enzymes involved in regulating DNA methylation (DNMT3, MBD4) is also increased in PCa and metastatic disease [74,186]. It is important to point out that global and gene specific hypomethylation changes are also associated with increased Gleason score and metastatic disease [161,187]. In Alzheimer’s disease, demethylase activity is affected by one-carbon metabolism (SAM:SAH ratio) [188], however, to our knowledge, similar studies have not been conducted in PCa.

Histone methylation changes are also common in PCa. Studies using immunohistochemical methods have reported an overexpression of H3K27me3 global levels in metastatic prostate tumors compared with non-malignant prostate tissues [72]. Although other histone methylation changes have been reported in PCa, changes in H3K27 methylation are receiving more attention since EZH2, the histone methyltransferase responsible for H3K27 methylation, is overexpressed during prostate tumorigenesis and is associated with biochemical recurrence in patients with PCa [78,80,189,190,191,192,193]. Upregulation of EZH2 is associated with repression of tumor suppressor genes, high proliferation rates, and increased tumor aggressiveness in PCa [78]. It is also directly involved in DNA methylation through interaction with DNA methyltransferases [190,194], and can target genes for de novo methylation in cancer [195]. Although this review focusses on processes that are affected by one-carbon metabolism, changes in demethylases are also relevant to PCa. Several reviews have been recently published [196,197,198].

### 6.2. Androgen Signaling Regulates the Expression and Activity of Methyltransferases in PCa

DNA and histone methyltransferases utilize SAM as substrate leading to SAH production, an inhibitor of methyltransferases [199]. Therefore, methylation reactions are largely dictated by the SAM:SAH ratio and the level of expression of the methyltransferases. For in depth coverage of the effect of SAM levels on activity and specificity of methyltransferases, the reader is referred to an excellent review by Mentch and Locasale [16].

**Figure 3:**
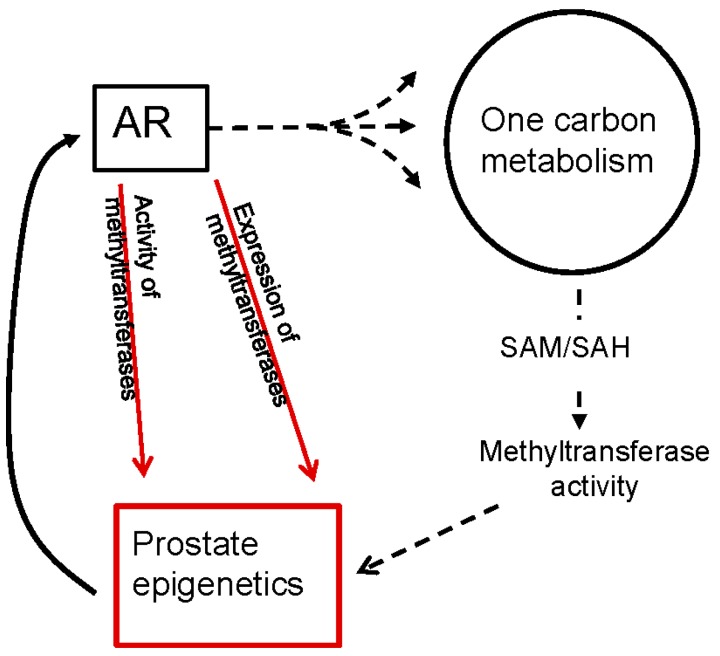
The impact of AR in prostate epigenetics. AR/androgen signaling can control the prostate epigenome: (1) indirectly by controlling expression of key enzymes involved in one-carbon metabolism and therefore the methylation potential of the cell (broken lines); or (2) directly by controlling the expression and activity of DNA and histone methyltransferases (solid lines).

The AR and androgen signaling play a role in controlling methylation, modulating the expression of methyltransferases and/or their activity. As discussed above, the AR has important roles in regulating GNMT and the metabolism of sarcosine and the enzymes involved in the diversion of methyl groups into the transsulfuration and polyamine synthesis pathways. Increased GNMT expression leads to increased levels of the methyltransferase inhibitor SAH [200]. Therefore, changes in AR activity indirectly affect methyltransferase activity by modulating the SAM:SAH ratio. In clinical samples of PCa, increased GNMT expression significantly correlates with high Gleason score and reduced disease-free survival [113]. These effects could partly be due to inhibition of DNA and histone methylation. Global DNA hypomethylation has been correlated with high Gleason score and metastatic PCa [161,187]. In addition to this indirect effect, the AR has a direct effect on methyltransferase activity by binding to these enzymes and, in some cases, promoting their recruitment into specific regions on the chromatin. For example, the AR interacts with and recruits EHZ2, increasing H3K27 methylation and epigenetic silencing and leading to oncogenic transformation [201,202]. Similarly, using the protein Menin as a bridge, the AR recruits MLL, a SET-like H3K4 histone methyltransferase [84], promoting AR-mediated transcription [203]. Interaction of the AR with demethylases has also been described. For example, JHDM2A, a H3K9 demethylase, binds to and is recruited to AR target genes upon androgen stimulation, resulting in H3K9 demethylation and transcriptional activation [204]. Similarly, the AR can directly interact with LSD1 on many AR-repressed genes. LSD1 is a lysine demethylase that has a repressive function by demethylating H3K4me1 and H3K4me2 in response to androgen [205]. Interestingly, the AR is a target for LSD1. Since DNA/chromatin methylation influences AR activity, these examples illustrate the fact that by modulating methyltransferase/demethylase activity and/or expression, the AR can also control its own expression and/or activity (Figure 3).

Changes in the one-carbon metabolism affecting methyltransferases (SAM:SAH ratio) also modulate DNA and chromatin methylation affecting the activity and/or expression of the AR. In addition, direct post-translational modification of the AR and/or co-activators by methyltransferases also occurs. The AR is directly methylated by the histone methyltransferase SET9 on lysine K632 resulting in enhanced transcriptional activity [206]. Interestingly, in CRPC, EZH2 functions as a coactivator for transcription factors including the AR. The activating function of EZH2 requires the methyltransferase domain, and it has been suggested that it functions by altering the AR-associated lysine methylation [207].

Finally, AR signaling can regulate expression of enzymes involved in histone methylation. It has been reported that androgens modulate expression of EZH2 in a concentration-dependent manner (EZH2 is repressed at 1 nM or higher). This effect requires a functional AR and is mediated by the binding of retinoblastoma (RB) and p130-associated proteins to the EZH2 promoter. While both mechanisms seem to be synergistic, their androgen dependence varies. RB-E2F1 are themselves regulated by androgens in PCa cells. p130 and its partner proteins bind to the EZH2 promoter in androgen-treated, but not in control treated cells [208,209]. Finally, expression of EZH2 can be repressed by miRNA101, which is regulated by androgens [210,211].

In summary, AR/androgen signaling has an important role in PCa epigenetics, both indirectly by controlling expression of key enzymes involved in one-carbon metabolism and associated pathways, and directly by controlling the expression and activity of DNA and histone methyltransferases. These effects can ultimately affect AR expression, which is also epigenetically controlled by DNA and histone methylation, or activity. These observations emphasize the precise link between the AR and one-carbon metabolism, and the potential effects that changes in AR signaling, that can occur with disease progression, may have on essential cellular processes (Figure 3).

## 7. Therapeutic Approaches to Prostate Cancer: Targeting the One-Carbon Metabolism

The accelerated proliferation of cancer cells places a robust demand on one-carbon metabolism, which can be exploited for anticancer therapies. The antifolate, aminopterin, originally used by Sydney Farber to treat pediatric patients with acute lymphoblastic leukemia, was the first successful anticancer chemotherapeutic agent [212]. Today, multiple drugs targeting enzymes within the folate cycle are FDA-approved to treat a variety of cancer types [15]; however, these drugs have had mixed reports for the treatment of PCa. While early studies indicated that the antifolate, MTX, might have been beneficial in the treatment of CRPC, subsequent studies failed to support the original findings [213,214,215]. Because AR inhibition during ADT decreases polyamine synthesis, which may in turn increase methyl group availability in the folate cycle, it has been suggested that MTX may be more beneficial in the treatment of PCa at earlier stages of the disease [63,160].

Other branches of the one-carbon metabolism network have been explored as therapeutic targets. As we discussed previously, the natural polyamines, putrescine, spermine and spermidine are ubiquitous molecules; however, their requirements are particularly high in rapidly growing tissues during normal growth and development, and in tumors [37,216,217,218]. Several reports have described increased polyamine levels in the blood and/or urine of cancer patients [219,220,221,222,223] and elevated levels correlate with more advanced disease and worse prognosis [216,224,225,226,227]. Increased polyamine levels are associated with increased cell proliferation, decreased apoptosis and increased expression of genes affecting tumor invasion and metastasis [37,228]. More recently, it has been shown that increased polyamine levels indirectly lead to immunosuppressive conditions facilitating tumor spread [228].

Changes in polyamine levels have been reported in PCa [143,218,225,229,230,231]. Underscoring the clinical relevance of polyamines to prostate cancer, preclinical data suggest that inhibition of polyamine synthesis blocks the progression of the disease [232,233,234,235,236,237]. All together these observations validate the polyamine pathway as chemopreventive and chemotherapeutic for PCa. Several trials have focused on targeting the polyamine pathway as a strategy for chemoprevention in patients at risk for aggressive PCa using difluoromethylornithine (DFMO), an inhibitor of ODC [238,239]. The results of those trials indicated that DMFO treatment results in decreased levels of putrescine, decreased rate of prostate growth, and a trend towards decreased PSA doubling time. A recent clinical trial demonstrated that DFMO caused nearly complete depletion of putrescine (97.6%) but not of spermidine and spermine (73.6% and 50.8%, respectively) [150], and while very well tolerated [143], it seemed to be largely ineffective as a chemotherapeutic agent. The lack of effectiveness could be in part due to compensatory mechanisms such as increased polyamine uptake from circulation, or upregulation of other enzymes involved in the pathway. Supporting this, it was shown that polyamine reduced diet induced or maintained the quality of life of patients with CRPC [151]. In addition, studies in cell lines and xenografts indicate increased efficacy when using DMFO in combination with polyamine transport inhibitors [234]. Increased levels of SAM-dc [142] and spermine synthase [150] have been observed in patients with PCa. Other pathway inhibitors including polyamine analogs [142,143,240] or SAM-dc inhibitors [143] have been previously pursued in clinical trials; however, they have demonstrated high toxicity or only partial responses.

In addition to drugs targeting one-carbon metabolism itself, methyltransferase inhibitors are also used to treat a variety of cancers, and several inhibitors are currently being investigated for cancer therapy [241]. 5′Azacitidine is a DNA methyltransferase inhibitor that is commonly used to treat myelodysplastic syndromes [242]. Importantly, epigenetic alterations, including DNA methylation, have been found to play an important role in therapy resistance, and 5′Azacitidine, and other demethylating agents, have been shown to be effective in combination therapy to improve chemosensitivity in other cancer types [243,244,245,246,247,248]. In PCa, for example, 5′Azacitidine improved chemosensitivity to docetaxel in patients with metastatic CRPC in phase I/II clinical trials [248].

Histone methyltransferases are also prime targets in epigenetic cancer therapy. EZH2, MLL, and DOT1L are potentially attractive targets in PCa, as all three modulate the activity of the AR [203,207,249]. MI-503 (MLL inhibitor) inhibits AR activity, and both DZNeP and MI-503 inhibit CRPC growth in mouse xenograft models [203,250]. Because EZH2 is overexpressed in metastatic CRPC and it drives a transcriptional signature that is associated with this stage of the disease, the potential use of EZH2 inhibitors in the treatment of CRPC is of particular interest [207,251]. Furthermore, EZH2 seems to have a role in both AR positive and AR negative CRPC, making EZH2 a versatile potential target in advanced PCa [207,251]. It is possible that therapeutics targeting one-carbon metabolism could work synergistically with direct methyltransferase inhibition to block the oncogenic functions of EZH2 and/or other methyltransferases in CRPC; however, this hypothesis remains to be tested.

## 8. Summary and Conclusions

The one-carbon metabolism network integrates several pathways that, together, play central roles in the biosynthesis of nucleic acids and lipids, amino acid and vitamin metabolism, the maintenance of redox status, methylation reactions and polyamine biogenesis. Because of the relevance of these pathways to cell growth and proliferation, they are critical not only for cellular homeostasis but also for tumorigenesis, and are therefore significant therapeutic targets.

**Figure 4:**
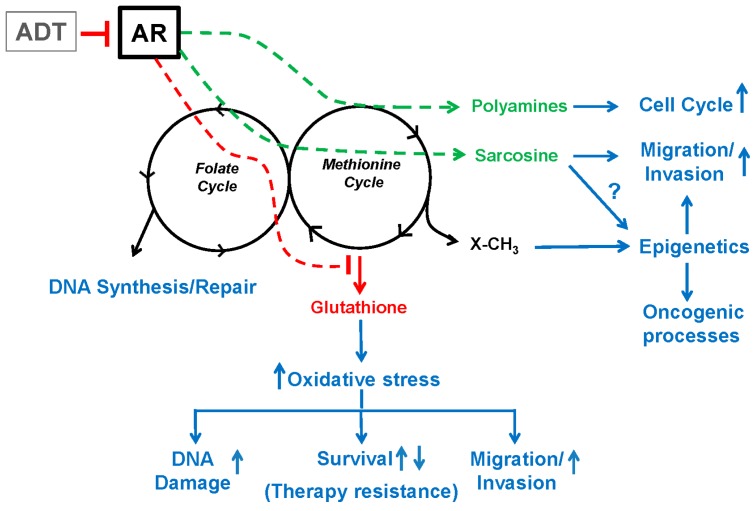
The AR impacts one-carbon metabolism and downstream processes by modulating the expression of specific associated enzymes. Green and red arrows/lines indicate reactions that are respectively activated and repressed by the AR via the modulation of enzyme expression. Enzymes include ODC1 (involved in polyamine synthesis), GNMT (catalyzes the conversion of glycine to sarcosine) and CBS (involved in glutathione synthesis). Metabolites increased and decreased by the AR in this manner are indicated in green and red, respectively. Black color depicts the one-carbon metabolism pathways and some other metabolites derived from it. Cellular processes potentially affected by AR-mediated one-carbon metabolism modulation are indicated in blue. By inhibiting AR, ADT (grey box) likely blocks many of these metabolic alterations in PCa cells, thus producing potentially beneficial and deleterious effects as discussed in the text.

The tight dependency among the pathways of the one-carbon metabolism network imposes an exquisite regulation to allow rapid responses to changes in cellular demands. The AR and androgen signaling regulate key enzymes involved in these pathways, including the ones that control the methylation potential of the cell and the entrance into the glutathione and polyamine biosynthetic pathways. Therefore, changes that occur in the AR levels or activity will have profound effects on the activity and output of the one-carbon metabolism network and downstream processes (Figure 4). ADT designed to decrease the levels of circulating androgens, or AR-directed therapies, are the mainstay treatments against advanced PCa, and are also used as adjuvants for local treatment of high risk disease. Because of their effect on AR signaling, these therapies affect the balance of the one-carbon metabolism. For example, it has been described that neo-adjuvant androgen blockade using an LHRH agonist, together with an anti-androgen, leads to decreased spermine and spermidine levels of the normal glands [158]. While in some instances the ADT-mediated effect on one-carbon metabolism may be beneficial, i.e., lowering the high levels of polyamines observed in cancer cells may help decrease their proliferative capacity, it is conceivable that it may also have a detrimental outcome. For instance, the blockade of polyamine synthesis would alter the flux of methyl groups toward other branches of the one-carbon metabolism network including the folate cycle, which potentially may lead to reduced sensitivity to the anti-folate methotrexate as discussed previously [63,160]. In addition, ADT or AR blockade would reduce the levels of GNMT, leading to increased SAM/SAH ratios and methyltransferase activity, a condition that maybe conducive to aggressive PCa (i.e., increased EZH2 levels in CRPC [78,191,193]). Finally, since the AR negatively regulates expression of CBS, an AR-signaling blockade would increase the flux towards the transsulfuration pathway, an effect that has been linked with increased therapeutic resistance [99,100,101]. Taken together these observations point to potential detrimental effects of ADT on one-carbon metabolism flux, and suggest that combination drug therapy in a precise order and timing may be helpful in the design of future clinical trials, and critical for successful treatment of PCa patients.

## Figures and Tables

**Table 1:** Androgen responsive genes of the one carbon metabolism network. * Data from the androgen responsive gene database (available online: http://argdb.fudan.edu.cn/index_info.php). MTHFD: methylenetetrahydrofolate dehydrogenase; SMS: spermine synthase; SAT: spermine/spermidine N1-acetyl transferase; CTH: cystathione gamma-lyase or gamma-cystathionase; GPX: glutathione peroxidase; GSR: glutathione reductase. Other names as in legend of Figure 1 and Figure 2.

Androgen Responsive Genes *			
Folate	Methionine	Polyamine	Transulfuration
SHMT	MAT	ODC	CBS
SARDH	AHCY	SMS	CTH
GLDC		SAT	GPX
DHFR		AMD1	GSR
MTHFD			
MTHFR			
GNMT			

## References

[1] Torre L.A., Bray F., Siegel R.L., Ferlay J., Lortet-Tieulent J., Jemal A. (2015). Global cancer statistics, 2012. CA Cancer J. Clin..

[2] De Winter R.J.A., Janssen P.J., Sleddens H.M., Verleun-Mooijman M.C., Trapman J., Brinkmann A.O., Santerse A.B., Schroder F.H., van der Kwast T.H. (1994). Androgen receptor status in localized and locally progressive hormone refractory human prostate cancer. Am. J. Pathol..

[3] Chodak G.W., Kranc D.M., Puy L.A., Takeda H., Johnson K., Chang C. (1992). Nuclear localization of androgen receptor in heterogeneous samples of normal, hyperplastic and neoplastic human prostate. J. Urol..

[4] Sadi M.V., Walsh P.C., Barrack E.R. (1991). Immunohistochemical study of androgen receptors in metastatic prostate cancer. Comparison of receptor content and response to hormonal therapy. Cancer.

[5] Hammerer P., Madersbacher S. (2012). Landmarks in hormonal therapy for prostate cancer. BJU Int..

[6] Scher H.I., Beer T.M., Higano C.S., Anand A., Taplin M.E., Efstathiou E., Rathkopf D., Shelkey J., Yu E.Y., Alumkal J. (2010). Antitumour activity of MDV3100 in castration-resistant prostate cancer: A phase 1–2 study. Lancet.

[7] Albertsen P.C., Hanley J.A., Fine J. (2005). 20-year outcomes following conservative management of clinically localized prostate cancer. JAMA.

[8] Merseburger A.S., Haas G.P., von Klot C.A. (2015). An update on enzalutamide in the treatment of prostate cancer. Ther. Adv. Urol..

[9] Pienta K.J., Bradley D. (2006). Mechanisms underlying the development of androgen-independent prostate cancer. Clin. Cancer Res..

[10] Sharma N.L., Massie C.E., Ramos-Montoya A., Zecchini V., Scott H.E., Lamb A.D., MacArthur S., Stark R., Warren A.Y., Mills I.G. (2013). The androgen receptor induces a distinct transcriptional program in castration-resistant prostate cancer in man. Cancer Cell.

[11] Wang Q., Li W., Zhang Y., Yuan X., Xu K., Yu J., Chen Z., Beroukhim R., Wang H., Lupien M. (2009). Androgen receptor regulates a distinct transcription program in androgen-independent prostate cancer. Cell.

[12] Barfeld S.J., Itkonen H.M., Urbanucci A., Mills I.G. (2014). Androgen-regulated metabolism and biosynthesis in prostate cancer. Endocr. Relat. Cancer.

[13] Massie C.E., Lynch A., Ramos-Montoya A., Boren J., Stark R., Fazli L., Warren A., Scott H., Madhu B., Sharma N. (2011). The androgen receptor fuels prostate cancer by regulating central metabolism and biosynthesis. EMBO J..

[14] Shafi A.A., Putluri V., Arnold J.M., Tsouko E., Maity S., Roberts J.M., Coarfa C., Frigo D.E., Putluri N., Sreekumar A. (2015). Differential regulation of metabolic pathways by androgen receptor (AR) and its constitutively active splice variant, AR-V7, in prostate cancer cells. Oncotarget.

[15] Locasale J.W. (2013). Serine, glycine and one-carbon units: Cancer metabolism in full circle. Nat. Rev. Cancer.

[16] Mentch S.J., Locasale J.W. (2016). One-carbon metabolism and epigenetics: Understanding the specificity. Ann. N. Y. Acad. Sci..

[17] Tibbetts A.S., Appling D.R. (2010). Compartmentalization of mammalian folate-mediated one-carbon metabolism. Annu. Rev. Nutr..

[18] Green T., Chen X., Ryan S., Asch A.S., Ruiz-Echevarria M.J. (2013). TMEFF2 and SARDH cooperate to modulate one-carbon metabolism and invasion of prostate cancer cells. Prostate.

[19] Khan A.P., Rajendiran T.M., Ateeq B., Asangani I.A., Athanikar J.N., Yocum A.K., Mehra R., Siddiqui J., Palapattu G., Wei J.T. (2013). The role of sarcosine metabolism in prostate cancer progression. Neoplasia.

[20] Sreekumar A., Poisson L.M., Rajendiran T.M., Khan A.P., Cao Q., Yu J., Laxman B., Mehra R., Lonigro R.J., Li Y. (2009). Metabolomic profiles delineate potential role for sarcosine in prostate cancer progression. Nature.

[21] Ottaviani S., Brooke G.N., O’Hanlon-Brown C., Waxman J., Ali S., Buluwela L. (2013). Characterisation of the androgen regulation of glycine *N*-methyltransferase in prostate cancer cells. J. Mol. Endocrinol..

[22] Johansson M., van Guelpen B., Vollset S.E., Hultdin J., Bergh A., Key T., Midttun O., Hallmans G., Ueland P.M., Stattin P. (2009). One-carbon metabolism and prostate cancer risk: Prospective investigation of seven circulating B vitamins and metabolites. Cancer Epidemiol. Biomark. Prev..

[23] Appling D.R. (1991). Compartmentation of folate-mediated one-carbon metabolism in eukaryotes. FASEB J..

[24] Fox J.T., Stover P.J. (2008). Folate-mediated one-carbon metabolism. Vitam. Horm..

[25] Scotti M., Stella L., Shearer E.J., Stover P.J. (2013). Modeling cellular compartmentation in one-carbon metabolism. Wiley Interdiscip. Rev. Syst. Biol. Med..

[26] Williams-Ashman H.G., Canellakis Z.N. (1979). Polyamines in mammalian biology and medicine. Perspect. Biol. Med..

[27] Saini P., Eyler D.E., Green R., Dever T.E. (2009). Hypusine-containing protein eIF5A promotes translation elongation. Nature.

[28] Stover P.J. (2009). One-carbon metabolism-genome interactions in folate-associated pathologies. J. Nutr..

[29] Luka Z. (2008). Methyltetrahydrofolate in folate-binding protein glycine *N*-methyltransferase. Vitam. Horm..

[30] Wagner C., Briggs W.T., Cook R.J. (1985). Inhibition of glycine *N*-methyltransferase activity by folate derivatives: Implications for regulation of methyl group metabolism. Biochem. Biophys. Res. Commun..

[31] Prudova A., Bauman Z., Braun A., Vitvitsky V., Lu S.C., Banerjee R. (2006). *S*-adenosylmethionine stabilizes cystathionine β-synthase and modulates redox capacity. Proc. Natl. Acad. Sci. USA.

[32] Pey A.L., Majtan T., Sanchez-Ruiz J.M., Kraus J.P. (2013). Human cystathionine β-synthase (CBS) contains two classes of binding sites for *S*-adenosylmethionine (SAM): Complex regulation of CBS activity and stability by SAM. Biochem. J..

[33] Bjelakovic G., Pavlovic D., Jevtovic T., Stojanovic I., Sokolovic D., Bjelakovic G.B., Nikolic J., Basic J. (2006). Vitamin B-12 and folic acid effects on polyamine metabolism in rat liver. Pteridines.

[34] Sun D., Wollin A., Stephen A.M. (2002). Moderate folate deficiency influences polyamine synthesis in rats. J. Nutr..

[35] Ott M., Gogvadze V., Orrenius S., Zhivotovsky B. (2007). Mitochondria, oxidative stress and cell death. Apoptosis.

[36] Ozben T. (2007). Oxidative stress and apoptosis: Impact on cancer therapy. J. Pharm. Sci..

[37] Gerner E.W., Meyskens F.L. (2004). Polyamines and cancer: Old molecules, new understanding. Nat. Rev. Cancer.

[38] Heby O. (1981). Role of polyamines in the control of cell proliferation and differentiation. Differentiation.

[39] Zhang W.C., Shyh-Chang N., Yang H., Rai A., Umashankar S., Ma S., Soh B.S., Sun L.L., Tai B.C., Nga M.E. (2012). Glycine decarboxylase activity drives non-small cell lung cancer tumor-initiating cells and tumorigenesis. Cell.

[40] Rahman L., Voeller D., Rahman M., Lipkowitz S., Allegra C., Barrett J.C., Kaye F.J., Zajac-Kaye M. (2004). Thymidylate synthase as an oncogene: A novel role for an essential DNA synthesis enzyme. Cancer Cell.

[41] Suzuki M., Tsukagoshi S., Saga Y., Ohwada M., Sato I. (1999). Enhanced expression of thymidylate synthase may be of prognostic importance in advanced cervical cancer. Oncology.

[42] Shintani Y., Ohta M., Hirabayashi H., Tanaka H., Iuchi K., Nakagawa K., Maeda H., Kido T., Miyoshi S., Matsuda H. (2003). New prognostic indicator for non-small-cell lung cancer, quantitation of thymidylate synthase by real-time reverse transcription polymerase chain reaction. Int. J. Cancer.

[43] Pestalozzi B.C., Peterson H.F., Gelber R.D., Goldhirsch A., Gusterson B.A., Trihia H., Lindtner J., Cortes-Funes H., Simmoncini E., Byrne M.J. (1997). Prognostic importance of thymidylate synthase expression in early breast cancer. J. Clin. Oncol..

[44] Nomura T., Nakagawa M., Fujita Y., Hanada T., Mimata H., Nomura Y. (2002). Clinical significance of thymidylate synthase expression in bladder cancer. Int. J. Urol..

[45] Mizutani Y., Wada H., Yoshida O., Fukushima M., Nonomura M., Nakao M., Miki T. (2003). Significance of thymidylate synthase activity in renal cell carcinoma. Clin. Cancer Res..

[46] Karlberg M., Ohrling K., Edler D., Hallstrom M., Ullen H., Ragnhammar P. (2010). Prognostic and predictive value of thymidylate synthase expression in primary colorectal cancer. Anticancer Res..

[47] Longley D.B., Harkin D.P., Johnston P.G. (2003). 5-Fluorouracil: Mechanisms of action and clinical strategies. Nat. Rev. Cancer.

[48] Zhang D., Wen X., Wu W., Guo Y., Cui W. (2015). Elevated homocysteine level and folate deficiency associated with increased overall risk of carcinogenesis: Meta-analysis of 83 case-control studies involving 35,758 individuals. PLoS ONE.

[49] Chen J., Gammon M.D., Chan W., Palomeque C., Wetmur J.G., Kabat G.C., Teitelbaum S.L., Britton J.A., Terry M.B., Neugut A.I. (2005). One-carbon metabolism, MTHFR polymorphisms, and risk of breast cancer. Cancer Res..

[50] Giovannucci E. (2002). Epidemiologic studies of folate and colorectal neoplasia: A review. J. Nutr..

[51] Chen P., Li C., Li X., Li J., Chu R., Wang H. (2014). Higher dietary folate intake reduces the breast cancer risk: A systematic review and meta-analysis. Br. J. Cancer.

[52] Kim Y.I., Pogribny I.P., Basnakian A.G., Miller J.W., Selhub J., James S.J., Mason J.B. (1997). Folate deficiency in rats induces DNA strand breaks and hypomethylation within the p53 tumor suppressor gene. Am. J. Clin. Nutr..

[53] Blount B.C., Mack M.M., Wehr C.M., MacGregor J.T., Hiatt R.A., Wang G., Wickramasinghe S.N., Everson R.B., Ames B.N. (1997). Folate deficiency causes uracil misincorporation into human DNA and chromosome breakage: Implications for cancer and neuronal damage. Proc. Natl. Acad. Sci. USA.

[54] Ergul E., Sazci A., Utkan Z., Canturk N.Z. (2003). Polymorphisms in the *MTHFR* gene are associated with breast cancer. Tumour Biol. J. Int. Soc. Oncodev. Biol. Med..

[55] Kumar P., Yadav U., Rai V. (2015). Methylenetetrahydrofolate reductase gene c677t polymorphism and breast cancer risk: Evidence for genetic susceptibility. Meta Gene.

[56] Lu Q., Jiang K., Li Q., Ji Y.J., Chen W.L., Xue X.H. (2015). Polymorphisms in the *MTHFR* gene are associated with breast cancer risk and prognosis in a chinese population. Tumour Biol. J. Int. Soc. Oncodev. Biol. Med..

[57] Maruti S.S., Ulrich C.M., Jupe E.R., White E. (2009). MTHFR C677T and postmenopausal breast cancer risk by intakes of one-carbon metabolism nutrients: A nested case-control study. Breast Cancer Res..

[58] Wang Y., Yang H., Duan G. (2015). Mthfr gene a1298c polymorphisms are associated with breast cancer risk among chinese population: Evidence based on an updated cumulative meta-analysis. Int. J. Clin. Exp. Med..

[59] Frosst P., Blom H.J., Milos R., Goyette P., Sheppard C.A., Matthews R.G., Boers G.J., den Heijer M., Kluijtmans L.A., van den Heuvel L.P. (1995). A candidate genetic risk factor for vascular disease: A common mutation in methylenetetrahydrofolate reductase. Nat. Genet..

[60] Bester A.C., Roniger M., Oren Y.S., Im M.M., Sarni D., Chaoat M., Bensimon A., Zamir G., Shewach D.S., Kerem B. (2011). Nucleotide deficiency promotes genomic instability in early stages of cancer development. Cell.

[61] Ehrlich M. (2009). DNA hypomethylation in cancer cells. Epigenomics.

[62] Poirier L.A. (1994). Methyl group deficiency in hepatocarcinogenesis. Drug Metab. Rev..

[63] Bistulfi G., Foster B.A., Karasik E., Gillard B., Miecznikowski J., Dhiman V.K., Smiraglia D.J. (2011). Dietary folate deficiency blocks prostate cancer progression in the tramp model. Cancer Prev. Res..

[64] Bistulfi G., Vandette E., Matsui S., Smiraglia D.J. (2010). Mild folate deficiency induces genetic and epigenetic instability and phenotype changes in prostate cancer cells. BMC Biol..

[65] Deghan Manshadi S., Ishiguro L., Sohn K.J., Medline A., Renlund R., Croxford R., Kim Y.I. (2014). Folic acid supplementation promotes mammary tumor progression in a rat model. PLoS ONE.

[66] Lindzon G.M., Medline A., Sohn K.J., Depeint F., Croxford R., Kim Y.I. (2009). Effect of folic acid supplementation on the progression of colorectal aberrant crypt foci. Carcinogenesis.

[67] Song J., Medline A., Mason J.B., Gallinger S., Kim Y.I. (2000). Effects of dietary folate on intestinal tumorigenesis in the apcmin mouse. Cancer Res..

[68] Kim Y.I. (2006). Folate: A magic bullet or a double edged sword for colorectal cancer prevention?. Gut.

[69] Ulrich C.M., Potter J.D. (2007). Folate and cancer—Timing is everything. JAMA.

[70] Greger V., Passarge E., Hopping W., Messmer E., Horsthemke B. (1989). Epigenetic changes may contribute to the formation and spontaneous regression of retinoblastoma. Hum. Genet..

[71] Yoo C.B., Jones P.A. (2006). Epigenetic therapy of cancer: Past, present and future. Nat. Rev. Drug Discov..

[72] Ellinger J., Kahl P., von der Gathen J., Heukamp L.C., Gütgemann I., Walter B., Hofstädter F., Bastian P.J., von Ruecker A., Müller S.C. (2012). Global histone H3K27 methylation levels are different in localized and metastatic prostate cancer. Cancer Investig..

[73] Barlesi F., Giaccone G., Gallegos-Ruiz M.I., Loundou A., Span S.W., Lefesvre P., Kruyt F.A., Rodriguez J.A. (2007). Global histone modifications predict prognosis of resected non small-cell lung cancer. J. Clin. Oncol..

[74] Bianco-Miotto T., Chiam K., Buchanan G., Jindal S., Day T.K., Thomas M., Pickering M.A., O’Loughlin M.A., Ryan N.K., Raymond W.A. (2010). Global levels of specific histone modifications and an epigenetic gene signature predict prostate cancer progression and development. Cancer Epidemiol. Biomark. Prev..

[75] Ellinger J., Kahl P., von der Gathen J., Rogenhofer S., Heukamp L.C., Gutgemann I., Walter B., Hofstadter F., Buttner R., Muller S.C. (2010). Global levels of histone modifications predict prostate cancer recurrence. Prostate.

[76] Seligson D.B., Horvath S., McBrian M.A., Mah V., Yu H., Tze S., Wang Q., Chia D., Goodglick L., Kurdistani S.K. (2009). Global levels of histone modifications predict prognosis in different cancers. Am. J. Pathol..

[77] Fraga M.F., Ballestar E., Villar-Garea A., Boix-Chornet M., Espada J., Schotta G., Bonaldi T., Haydon C., Ropero S., Petrie K. (2005). Loss of acetylation at LYS16 and trimethylation at LYS20 of histone H4 is a common hallmark of human cancer. Nat. Genet..

[78] Bachmann I.M., Halvorsen O.J., Collett K., Stefansson I.M., Straume O., Haukaas S.A., Salvesen H.B., Otte A.P., Akslen L.A. (2006). EZH2 expression is associated with high proliferation rate and aggressive tumor subgroups in cutaneous melanoma and cancers of the endometrium, prostate, and breast. J. Clin. Oncol..

[79] Simon J.A., Lange C.A. (2008). Roles of the EZH2 histone methyltransferase in cancer epigenetics. Mutat. Res..

[80] Van Leenders G.J.L.H., Dukers D., Hessels D., van den Kieboom S.W.M., Hulsbergen C.A., Witjes J.A., Otte A.P., Meijer C.J., Raaphorst F.M. (2007). Polycomb-group oncogenes EZH2, BMI1, and RING1 are overexpressed in prostate cancer with adverse pathologic and clinical features. Eur. Urol..

[81] Hyland P.L., McDade S.S., McCloskey R., Dickson G.J., Arthur K., McCance D.J., Patel D. (2011). Evidence for alteration of EZH2, BMI1, and KDM6A and epigenetic reprogramming in human papillomavirus type 16 E6/E7-expressing keratinocytes. J. Virol..

[82] Lee J.Y., Kong G. (2015). Dot1l: A new therapeutic target for aggressive breast cancer. Oncotarget.

[83] Wong M., Polly P., Liu T. (2015). The histone methyltransferase DOT1L: Regulatory functions and a cancer therapy target. Am. J. Cancer Res..

[84] Dou Y., Hess J.L. (2008). Mechanisms of transcriptional regulation by MLL and its disruption in acute leukemia. Int. J. Hematol..

[85] Krivtsov A.V., Armstrong S.A. (2007). MLL translocations, histone modifications and leukaemia stem-cell development. Nat. Rev. Cancer.

[86] Orzan F., Pellegatta S., Poliani P.L., Pisati F., Caldera V., Menghi F., Kapetis D., Marras C., Schiffer D., Finocchiaro G. (2011). Enhancer of zeste 2 (EZH2) is up-regulated in malignant gliomas and in glioma stem-like cells. Neuropathol. Appl. Neurobiol..

[87] Kerr S.J. (1972). Competing methyltransferase systems. J. Biol. Chem..

[88] Shyh-Chang N., Locasale J.W., Lyssiotis C.A., Zheng Y., Teo R.Y., Ratanasirintrawoot S., Zhang J., Onder T., Unternaehrer J.J., Zhu H. (2013). Influence of threonine metabolism on *S*-adenosylmethionine and histone methylation. Science.

[89] Huang W.Y., Yang P.M., Chang Y.F., Marquez V.E., Chen C.C. (2011). Methotrexate induces apoptosis through p53/p21-dependent pathway and increases e-cadherin expression through downregulation of HDAC/EZH2. Biochem. Pharmacol..

[90] Mandal S., Mandal A., Johansson H.E., Orjalo A.V., Park M.H. (2013). Depletion of cellular polyamines, spermidine and spermine, causes a total arrest in translation and growth in mammalian cells. Proc. Natl. Acad. Sci. USA.

[91] O’Brien T.G., Megosh L.C., Gilliard G., Soler A.P. (1997). Ornithine decarboxylase overexpression is a sufficient condition for tumor promotion in mouse skin. Cancer Res..

[92] Nilsson J.A., Keller U.B., Baudino T.A., Yang C., Norton S., Old J.A., Nilsson L.M., Neale G., Kramer D.L., Porter C.W. (2005). Targeting ornithine decarboxylase in MYC-induced lymphomagenesis prevents tumor formation. Cancer Cell.

[93] Lee O.J., Schneider-Stock R., McChesney P.A., Kuester D., Roessner A., Vieth M., Moskaluk C.A., El-Rifai W. (2005). Hypermethylation and loss of expression of glutathione peroxidase-3 in barrett’s tumorigenesis. Neoplasia.

[94] Zhang X., Yang J.J., Kim Y.S., Kim K.Y., Ahn W.S., Yang S. (2010). An 8-gene signature, including methylated and down-regulated glutathione peroxidase 3, of gastric cancer. Int. J. Oncol..

[95] Yu Y.P., Yu G., Tseng G., Cieply K., Nelson J., Defrances M., Zarnegar R., Michalopoulos G., Luo J.H. (2007). Glutathione peroxidase 3, deleted or methylated in prostate cancer, suppresses prostate cancer growth and metastasis. Cancer Res..

[96] Peng D.F., Razvi M., Chen H., Washington K., Roessner A., Schneider-Stock R., El-Rifai W. (2009). DNA hypermethylation regulates the expression of members of the Mu-class glutathione *S*-transferases and glutathione peroxidases in barrett’s adenocarcinoma. Gut.

[97] Jhaveri M.S., Morrow C.S. (1998). Methylation-mediated regulation of the glutathione *S*-transferase p1 gene in human breast cancer cells. Gene.

[98] Lee W.H., Morton R.A., Epstein J.I., Brooks J.D., Campbell P.A., Bova G.S., Hsieh W.S., Isaacs W.B., Nelson W.G. (1994). Cytidine methylation of regulatory sequences near the Pi-class glutathione *S*-transferase gene accompanies human prostatic carcinogenesis. Proc. Natl. Acad. Sci. USA.

[99] Townsend D.M., Tew K.D. (2003). The role of glutathione-*S*-transferase in anti-cancer drug resistance. Oncogene.

[100] Godwin A.K., Meister A., O’Dwyer P.J., Huang C.S., Hamilton T.C., Anderson M.E. (1992). High resistance to cisplatin in human ovarian cancer cell lines is associated with marked increase of glutathione synthesis. Proc. Natl. Acad. Sci. USA.

[101] Kramer R.A., Zakher J., Kim G. (1988). Role of the glutathione redox cycle in acquired and de novo multidrug resistance. Science.

[102] Chen N., Liu Y., Greiner C.D., Holtzman J.L. (2000). Physiologic concentrations of homocysteine inhibit the human plasma gsh peroxidase that reduces organic hydroperoxides. J. Lab. Clin. Med..

[103] Durmaz A., Dikmen N. (2007). Homocysteine effects on cellular glutathione peroxidase (GPX-1) activity under in vitro conditions. J. Enzym. Inhib. Med. Chem..

[104] Handy D.E., Zhang Y., Loscalzo J. (2005). Homocysteine down-regulates cellular glutathione peroxidase (GPX1) by decreasing translation. J. Biol. Chem..

[105] Tastekin D., Erturk K., Bozbey H.U., Olmuscelik O., Kiziltan H., Tuna S., Tas F. (2015). Plasma homocysteine, folate and vitamin B12 levels in patients with lung cancer. Exp. Oncol..

[106] Lubos E., Loscalzo J., Handy D.E. (2007). Homocysteine and glutathione peroxidase-1. Antioxid. Redox Signal..

[107] Lin J., Lee I.M., Song Y., Cook N.R., Selhub J., Manson J.E., Buring J.E., Zhang S.M. (2010). Plasma homocysteine and cysteine and risk of breast cancer in women. Cancer Res..

[108] Matsumoto T., Sakari M., Okada M., Yokoyama A., Takahashi S., Kouzmenko A., Kato S. (2013). The androgen receptor in health and disease. Annu. Rev. Physiol..

[109] Tsouko E., Khan A.S., White M.A., Han J.J., Shi Y., Merchant F.A., Sharpe M.A., Xin L., Frigo D.E. (2014). Regulation of the pentose phosphate pathway by an androgen receptor-mtor-mediated mechanism and its role in prostate cancer cell growth. Oncogenesis.

[110] Tennakoon J.B., Shi Y., Han J.J., Tsouko E., White M.A., Burns A.R., Zhang A., Xia X., Ilkayeva O.R., Xin L. (2014). Androgens regulate prostate cancer cell growth via an AMPK-PGC-1α-mediated metabolic switch. Oncogene.

[111] Luka Z., Mudd S.H., Wagner C. (2009). Glycine *N*-methyltransferase and regulation of *S*-adenosylmethionine levels. J. Biol. Chem..

[112] Huang Y.C., Lee C.M., Chen M., Chung M.Y., Chang Y.H., Huang W.J., Ho D.M., Pan C.C., Wu T.T., Yang S. (2007). Haplotypes, loss of heterozygosity, and expression levels of glycine *N*-methyltransferase in prostate cancer. Clin. Cancer Res..

[113] Song Y.H., Shiota M., Kuroiwa K., Naito S., Oda Y. (2011). The important role of glycine *N*-methyltransferase in the carcinogenesis and progression of prostate cancer. Mod. Pathol..

[114] Putluri N., Shojaie A., Vasu V.T., Nalluri S., Vareed S.K., Putluri V., Vivekanandan-Giri A., Byun J., Pennathur S., Sana T.R. (2011). Metabolomic profiling reveals a role for androgen in activating amino acid metabolism and methylation in prostate cancer cells. PLoS ONE.

[115] Yoon J.K., Kim D.H., Koo J.S. (2014). Implications of differences in expression of sarcosine metabolism-related proteins according to the molecular subtype of breast cancer. J. Transl. Med..

[116] Ulanovskaya O.A., Zuhl A.M., Cravatt B.F. (2013). NNMT promotes epigenetic remodeling in cancer by creating a metabolic methylation sink. Nat. Chem. Biol..

[117] Zhang J., Wang Y., Li G., Yu H., Xie X. (2014). Down-regulation of nicotinamide *N*-methyltransferase induces apoptosis in human breast cancer cells via the mitochondria-mediated pathway. PLoS ONE.

[118] Chen X., Overcash R., Green T., Hoffman D., Asch A.S., Ruiz-Echevarria M.J. (2011). The tumor suppressor activity of the transmembrane protein with epidermal growth factor and two follistatin motifs 2 (TMEFF2) correlates with its ability to modulate sarcosine levels. J. Biol. Chem..

[119] Corbin J.M., Overcash R.F., Wren J.D., Coburn A., Tipton G.J., Ezzell J.A., McNaughton K.K., Fung K.M., Kosanke S.D., Ruiz-Echevarria M.J. (2016). Analysis of TMEFF2 allografts and transgenic mouse models reveals roles in prostate regeneration and cancer. Prostate.

[120] Reed M.C., Thomas R.L., Pavisic J., James S.J., Ulrich C.M., Nijhout H.F. (2008). A mathematical model of glutathione metabolism. Theor. Biol. Med. Model..

[121] Li J.J., Li Q., Du H.P., Wang Y.L., You S.J., Wang F., Xu X.S., Cheng J., Cao Y.J., Liu C.F. (2015). Homocysteine triggers inflammatory responses in macrophages through inhibiting CSE-H2s signaling via DNA hypermethylation of CSE promoter. Int. J. Mol. Sci..

[122] Kabil O., Banerjee R. (2014). Enzymology of h2s biogenesis, decay and signaling. Antioxid. Redox Signal..

[123] Kajimura M., Fukuda R., Bateman R.M., Yamamoto T., Suematsu M. (2010). Interactions of multiple gas-transducing systems: Hallmarks and uncertainties of Co, No, and H_2_S gas biology. Antioxid. Redox Signal..

[124] Mustafa A.K., Gadalla M.M., Snyder S.H. (2009). Signaling by gasotransmitters. Sci. Signal..

[125] Zhao K., Li S., Wu L., Lai C., Yang G. (2014). Hydrogen sulfide represses androgen receptor transactivation by targeting at the second zinc finger module. J. Biol. Chem..

[126] Schalinske K.L., Smazal A.L. (2012). Homocysteine imbalance: A pathological metabolic marker. Adv. Nutr..

[127] Bhattacharyya S., Saha S., Giri K., Lanza I.R., Nair K.S., Jennings N.B., Rodriguez-Aguayo C., Lopez-Berestein G., Basal E., Weaver A.L. (2013). Cystathionine β-synthase (CBS) contributes to advanced ovarian cancer progression and drug resistance. PLoS ONE.

[128] Szabo C., Coletta C., Chao C., Módis K., Szczesny B., Papapetropoulos A., Hellmich M.R. (2013). Tumor-derived hydrogen sulfide, produced by cystathionine-β-synthase, stimulates bioenergetics, cell proliferation, and angiogenesis in colon cancer. Proc. Natl. Acad. Sci. USA.

[129] Janošík M., Kery V., Gaustadnes M., Maclean K.N., Kraus J.P. (2001). Regulation of human cystathionine β-synthase by *S*-adenosyl-l-methionine: Evidence for two catalytically active conformations involving an autoinhibitory domain in the C-terminal region. Biochemistry.

[130] Prudova A., Albin M., Bauman Z., Lin A., Vitvitsky V., Banerjee R. (2007). Testosterone regulation of homocysteine metabolism modulates redox status in human prostate cancer cells. Antioxid. Redox Signal..

[131] Guo H., Gai J.-W., Wang Y., Jin H.-F., Du J.-B., Jin J. (2012). Characterization of hydrogen sulfide and its synthases, cystathionine β-synthase and cystathionine γ-lyase, in human prostatic tissue and cells. Urology.

[132] Zhang W., Braun A., Bauman Z., Olteanu H., Madzelan P., Banerjee R. (2005). Expression profiling of homocysteine junction enzymes in the nci60 panel of human cancer cell lines. Cancer Res..

[133] Al-Awadi F., Yang M., Tan Y., Han Q., Li S., Hoffman R.M. (2008). Human tumor growth in nude mice is associated with decreased plasma cysteine and homocysteine. Anticancer Res..

[134] Stabler S., Koyama T., Zhao Z., Martinez-Ferrer M., Allen R.H., Luka Z., Loukachevitch L.V., Clark P.E., Wagner C., Bhowmick N.A. (2011). Serum methionine metabolites are risk factors for metastatic prostate cancer progression. PLoS ONE.

[135] Chwatko G., Forma E., Wilkosz J., Głowacki R., Jóźwiak P., Różański W., Bryś M., Krześlak A. (2013). Thiosulfate in urine as a facilitator in the diagnosis of prostate cancer for patients with prostate-specific antigen less or equal 10 ng/mL. Clin. Chem. Lab. Med..

[136] Hatayama I., Satoh K., Sato K. (1986). Developmental and hormonal regulation of the major form of hepatic glutathione s-transferase in male mice. Biochem. Biophys. Res. Commun..

[137] Ikeda H., Serria M.S., Kakizaki I., Hatayama I., Satoh K., Tsuchida S., Muramatsu M., Nishi S., Sakai M. (2002). Activation of mouse pi-class glutathione *S*-transferase gene by Nrf2(Nf-E2-related factor 2) and androgen. Biochem. J..

[138] Imperlini E., Mancini A., Spaziani S., Martone D., Alfieri A., Gemei M., del Vecchio L., Buono P., Orru S. (2010). Androgen receptor signaling induced by supraphysiological doses of dihydrotestosterone in human peripheral blood lymphocytes. Proteomics.

[139] Khandrika L., Kumar B., Koul S., Maroni P., Koul H.K. (2009). Oxidative stress in prostate cancer. Cancer Lett..

[140] Pendeville H., Carpino N., Marine J.C., Takahashi Y., Muller M., Martial J.A., Cleveland J.L. (2001). The ornithine decarboxylase gene is essential for cell survival during early murine development. Mol. Cell. Biol..

[141] Casero R.A., Pegg A.E. (2009). Polyamine catabolism and disease. Biochem. J..

[142] Nowotarski S.L., Woster P.M., Casero R.A. (2013). Polyamines and cancer: Implications for chemotherapy and chemoprevention. Expert Rev. Mol. Med..

[143] Casero R.A., Marton L.J. (2007). Targeting polyamine metabolism and function in cancer and other hyperproliferative diseases. Nat. Rev. Drug Discov..

[144] Giardiello F.M., Hamilton S.R., Hylind L.M., Yang V.W., Tamez P., Casero R.A. (1997). Ornithine decarboxylase and polyamines in familial adenomatous polyposis. Cancer Res..

[145] Pegg A.E., Lockwood D.H., Williams-Ashman H.G. (1970). Concentrations of putrescine and polyamines and their enzymic synthesis during androgen-induced prostatic growth. Biochem. J..

[146] Blackshear P.J., Manzella J.M., Stumpo D.J., Wen L., Huang J.K., Oyen O., Young W.S. (1989). High level, cell-specific expression of ornithine decarboxylase transcripts in rat genitourinary tissues. Mol. Endocrinol..

[147] Pegg A.E. (1988). Polyamine metabolism and its importance in neoplastic growth and a target for chemotherapy. Cancer Res..

[148] Janne J., Alhonen L., Leinonen P. (1991). Polyamines: From molecular biology to clinical applications. Ann. Med..

[149] Mohan R.R., Challa A., Gupta S., Bostwick D.G., Ahmad N., Agarwal R., Marengo S.R., Amini S.B., Paras F., MacLennan G.T. (1999). Overexpression of ornithine decarboxylase in prostate cancer and prostatic fluid in humans. Clin. Cancer Res..

[150] Rhodes D.R., Barrette T.R., Rubin M.A., Ghosh D., Chinnaiyan A.M. (2002). Meta-analysis of microarrays: Interstudy validation of gene expression profiles reveals pathway dysregulation in prostate cancer. Cancer Res..

[151] Cipolla B.G., Havouis R., Moulinoux J.P. (2010). Polyamine reduced diet (PRD) nutrition therapy in hormone refractory prostate cancer patients. Biomed. Pharmacother..

[152] Janne O.A., Crozat A., Palvimo J., Eisenberg L.M. (1991). Androgen-regulation of ornithine decarboxylase and *S*-adenosylmethionine decarboxylase genes. J. Steroid Biochem. Mol. Biol..

[153] Fjosne H.E., Strand H., Sunde A. (1992). Dose-dependent induction of ornithine decarboxylase and *S*-adenosyl-methionine decarboxylase activity by testosterone in the accessory sex organs of male rats. Prostate.

[154] Cyriac J., Haleem R., Cai X., Wang Z. (2002). Androgen regulation of spermidine synthase expression in the rat prostate. Prostate.

[155] Crozat A., Palvimo J.J., Julkunen M., Janne O.A. (1992). Comparison of androgen regulation of ornithine decarboxylase and *S*-adenosylmethionine decarboxylase gene expression in rodent kidney and accessory sex organs. Endocrinology.

[156] Fjosne H.E., Strand H., Ostensen M.A., Sunde A. (1988). Ornithine decarboxylase and *S*-adenosylmethionine decarboxylase activity in the accessory sex organs of intact, castrated, and androgen-stimulated castrated rats. Prostate.

[157] Bai G., Kasper S., Matusik R.J., Rennie P.S., Moshier J.A., Krongrad A. (1998). Androgen regulation of the human ornithine decarboxylase promoter in prostate cancer cells. J. Androl..

[158] Cohen R.J., Fujiwara K., Holland J.W., McNeal J.E. (2001). Polyamines in prostatic epithelial cells and adenocarcinoma: The effects of androgen blockade. Prostate.

[159] Lloyd S.M., Arnold J., Sreekumar A. (2015). Metabolomic profiling of hormone-dependent cancers: A bird’s eye view. Trends Endocrinol. Metab..

[160] Bistulfi G., Diegelman P., Foster B.A., Kramer D.L., Porter C.W., Smiraglia D.J. (2009). Polyamine biosynthesis impacts cellular folate requirements necessary to maintain *S*-adenosylmethionine and nucleotide pools. FASEB J..

[161] Yegnasubramanian S., Haffner M.C., Zhang Y., Gurel B., Cornish T.C., Wu Z., Irizarry R.A., Morgan J., Hicks J., DeWeese T.L. (2008). DNA hypomethylation arises later in prostate cancer progression than CPG island hypermethylation and contributes to metastatic tumor heterogeneity. Cancer Res..

[162] Jerónimo C., Bastian P.J., Bjartell A., Carbone G.M., Catto J.W.F., Clark S.J., Henrique R., Nelson W.G., Shariat S.F. (2011). Epigenetics in prostate cancer: Biologic and clinical relevance. Eur. Urol..

[163] Labbe D.P., Zadra G., Ebot E.M., Mucci L.A., Kantoff P.W., Loda M., Brown M. (2015). Role of diet in prostate cancer: The epigenetic link. Oncogene.

[164] Seligson D.B., Horvath S., Shi T., Yu H., Tze S., Grunstein M., Kurdistani S.K. (2005). Global histone modification patterns predict risk of prostate cancer recurrence. Nature.

[165] Perry A.S., Watson R.W., Lawler M., Hollywood D. (2010). The epigenome as a therapeutic target in prostate cancer. Nat. Rev. Urol..

[166] Nelson W.G., de Marzo A.M., Yegnasubramanian S. (2009). Epigenetic alterations in human prostate cancers. Endocrinology.

[167] Yegnasubramanian S., Kowalski J., Gonzalgo M.L., Zahurak M., Piantadosi S., Walsh P.C., Bova G.S., de Marzo A.M., Isaacs W.B., Nelson W.G. (2004). Hypermethylation of CPG islands in primary and metastatic human prostate cancer. Cancer Res..

[168] Jeronimo C., Henrique R., Hoque M.O., Mambo E., Ribeiro F.R., Varzim G., Oliveira J., Teixeira M.R., Lopes C., Sidransky D. (2004). A quantitative promoter methylation profile of prostate cancer. Clin. Cancer Res..

[169] Maruyama R., Toyooka S., Toyooka K.O., Virmani A.K., Zöchbauer-Müller S., Farinas A.J., Minna J.D., McConnell J., Frenkel E.P., Gazdar A.F. (2002). Aberrant promoter methylation profile of prostate cancers and its relationship to clinicopathological features. Clin. Cancer Res..

[170] Florl A.R., Steinhoff C., Müller M., Seifert H.H., Hader C., Engers R., Ackermann R., Schulz W.A. (2004). Coordinate hypermethylation at specific genes in prostate carcinoma precedes line-1 hypomethylation. Br. J. Cancer.

[171] Padar A., Sathyanarayana U.G., Suzuki M., Maruyama R., Hsieh J.T., Frenkel E.P., Minna J.D., Gazdar A.F. (2003). Inactivation of cyclin D2 gene in prostate cancers by aberrant promoter methylation. Clin. Cancer Res..

[172] Yamanaka M., Watanabe M., Yamada Y., Takagi A., Murata T., Takahashi H., Suzuki H., Ito H., Tsukino H., Katoh T. (2003). Altered methylation of multiple genes in carcinogenesis of the prostate. Int. J. Cancer.

[173] Ellinger J., Bastian P.J., Jurgan T., Biermann K., Kahl P., Heukamp L.C., Wernert N., Müller S.C., von Ruecker A. (2008). Cpg island hypermethylation at multiple gene sites in diagnosis and prognosis of prostate cancer. Urology.

[174] Lodygin D., Epanchintsev A., Menssen A., Diebold J., Hermeking H. (2005). Functional epigenomics identifies genes frequently silenced in prostate cancer. Cancer Res..

[175] Perry A.S., Foley R., Woodson K., Lawler M. (2006). The emerging roles of DNA methylation in the clinical management of prostate cancer. Endocr. Relat. Cancer.

[176] Li L.C. (2007). Epigenetics of prostate cancer. Front. Biosci. J. Virtual Libr..

[177] Sasaki M., Tanaka Y., Perinchery G., Dharia A., Kotcherguina I., Fujimoto S., Dahiya R. (2002). Methylation and inactivation of estrogen, progesterone, and androgen receptors in prostate cancer. J. Natl. Cancer Inst..

[178] Reibenwein J., Pils D., Horak P., Tomicek B., Goldner G., Worel N., Elandt K., Krainer M. (2007). Promoter hypermethylation of GSTP1, AR, and 14–3-3σ in serum of prostate cancer patients and its clinical relevance. Prostate.

[179] Chen M.F., Chen W.C., Chang Y.J., Wu C.F., Wu C.T. (2010). Role of DNA methyltransferase 1 in hormone-resistant prostate cancer. J. Mol. Med..

[180] Patra S.K., Patra A., Zhao H., Dahiya R. (2002). DNA methyltransferase and demethylase in human prostate cancer. Mol. Carcinog..

[181] Zhang W., Jiao H., Zhang X., Zhao R., Wang F., He W., Zong H., Fan Q., Wang L. (2015). Correlation between the expression of DNMT1, and GSTP1 and APC, and the methylation status of GSTP1 and APC in association with their clinical significance in prostate cancer. Mol. Med. Rep..

[182] Valdez C.D., Kunju L., Daignault S., Wojno K.J., Day M.L. (2013). The e2f1/dnmt1 axis is associated with the development of ar negative castration resistant prostate cancer. Prostate.

[183] Zhang Q., Chen L., Helfand B.T., Jang T.L., Sharma V., Kozlowski J., Kuzel T.M., Zhu L.J., Yang X.J., Javonovic B. (2011). Tgf-beta regulates DNA methyltransferase expression in prostate cancer, correlates with aggressive capabilities, and predicts disease recurrence. PLoS ONE.

[184] Kinney S.R., Moser M.T., Pascual M., Greally J.M., Foster B.A., Karpf A.R. (2010). Opposing roles of DNMT1 in early- and late-stage murine prostate cancer. Mol. Cell. Biol..

[185] McCabe M.T., Low J.A., Daignault S., Imperiale M.J., Wojno K.J., Day M.L. (2006). Inhibition of DNA methyltransferase activity prevents tumorigenesis in a mouse model of prostate cancer. Cancer Res..

[186] Kobayashi Y., Absher D.M., Gulzar Z.G., Young S.R., McKenney J.K., Peehl D.M., Brooks J.D., Myers R.M., Sherlock G. (2011). DNA methylation profiling reveals novel biomarkers and important roles for DNA methyltransferases in prostate cancer. Genome Res..

[187] Brothman A.R., Swanson G., Maxwell T.M., Cui J., Murphy K.J., Herrick J., Speights V.O., Isaac J., Rohr L.R. (2005). Global hypomethylation is common in prostate cancer cells: A quantitative predictor for clinical outcome?. Cancer Gen. Cytogenet..

[188] Fuso A., Nicolia V., Cavallaro R.A., Scarpa S. (2011). DNA methylase and demethylase activities are modulated by one-carbon metabolism in Alzheimer’s disease models. J. Nut. Biochem..

[189] Rhodes D.R., Sanda M.G., Otte A.P., Chinnaiyan A.M., Rubin M.A. (2003). Multiplex biomarker approach for determining risk of prostate-specific antigen-defined recurrence of prostate cancer. J. Natl. Cancer Inst..

[190] Hoffmann M.J., Engers R., Florl A.R., Otte A.P., Müller M., Schulz W.A. (2007). Expression changes in EZH2, but not in BMI-1, SIRT1, DNMT1 or DNMT3b, are associated with DNA methylation changes in prostate cancer. Cancer Biol. Ther..

[191] Varambally S., Dhanasekaran S.M., Zhou M., Barrette T.R., Kumar-Sinha C., Sanda M.G., Ghosh D., Pienta K.J., Sewalt R.G.A.B., Otte A.P. (2002). The polycomb group protein EZH2 is involved in progression of prostate cancer. Nature.

[192] Laitinen S., Martikainen P.M., Tolonen T., Isola J., Tammela T.L.J., Visakorpi T. (2008). EZH2, Ki-67 and MCM7 are prognostic markers in prostatectomy treated patients. Int. J. Cancer.

[193] Yu J., Rhodes D.R., Tomlins S.A., Cao X., Chen G., Mehra R., Wang X., Ghosh D., Shah R.B. (2007). A polycomb repression signature in metastatic prostate cancer predicts cancer outcome. Cancer Res..

[194] Viré E., Brenner C., Deplus R., Blanchon L., Fraga M., Didelot C., Morey L., van Eynde A., Bernard D., Vanderwinden J.M. (2006). The polycomb group protein EZH2 directly controls DNA methylation. Nature.

[195] Schlesinger Y., Straussman R., Keshet I., Farkash S., Hecht M., Zimmerman J., Eden E., Yakhini Z., Ben-Shushan E., Reubinoff B.E. (2007). Polycomb-mediated methylation on LYS27 of histone H3 pre-marks genes for de novo methylation in cancer. Nat. Genet..

[196] Crea F., Sun L., Mai A., Chiang Y.T., Farrar W.L., Danesi R., Helgason C.D. (2012). The emerging role of histone lysine demethylases in prostate cancer. Mol. Cancer.

[197] Berry W.L., Janknecht R. (2013). KDM4/JMJD2 histone demethylases: Epigenetic regulators in cancer cells. Cancer Res..

[198] Franci G., Ciotta A., Altucci L. (2014). The Jumonji family: Past, present and future of histone demethylases in cancer. Biomol. Concepts.

[199] Deguchi T., Barchas J. (1971). Inhibition of transmethylations of biogenic amines by *S*-adenosylhomocysteine. Enhancement of transmethylation by adenosylhomocysteinase. J. Biol. Chem..

[200] Wang Y.-C., Tang F.-Y., Chen S.-Y., Chen Y.-M., Chiang E.-P.I. (2011). Glycine-N methyltransferase expression in HepG2 cells is involved in methyl group homeostasis by regulating transmethylation kinetics and DNA methylation. J. Nutr..

[201] Zhao J.C., Yu J., Runkle C., Wu L., Hu M., Wu D., Liu J.S., Wang Q., Qin Z.S., Yu J. (2012). Cooperation between polycomb and androgen receptor during oncogenic transformation. Genome Res..

[202] Chng K.R., Chang C.W., Tan S.K., Yang C., Hong S.Z., Sng N.Y., Cheung E. (2012). A transcriptional repressor co-regulatory network governing androgen response in prostate cancers. EMBO J..

[203] Malik R., Khan A.P., Asangani I.A., Cieślik M., Prensner J.R., Wang X., Iyer M.K., Jiang X., Borkin D., Escara-Wilke J. (2015). Targeting the MLL complex in castration resistant prostate cancer. Nat. Med..

[204] Yamane K., Toumazou C., Tsukada Y., Erdjument-Bromage H., Tempst P., Wong J., Zhang Y. (2006). JHDM2A, a JMJC-containing H3K9 demethylase, facilitates transcription activation by androgen receptor. Cell.

[205] Cai C., He H.H., Chen S., Coleman I., Wang H., Fang Z., Chen S., Nelson P.S., Liu X.S., Brown M. (2011). Androgen receptor gene expression in prostate cancer is directly suppressed by the androgen receptor through recruitment of lysine-specific demethylase 1. Cancer Cell.

[206] Gaughan L., Stockley J., Wang N., McCracken S.R.C., Treumann A., Armstrong K., Shaheen F., Watt K., McEwan I.J., Wang C. (2011). Regulation of the androgen receptor by set9-mediated methylation. Nucleic Acids Res..

[207] Xu K., Wu Z.J., Groner A.C., He H.H., Cai C., Lis R.T., Wu X., Stack E.C., Loda M., Liu T. (2012). Ezh2 oncogenic activity in castration-resistant prostate cancer cells is polycomb-independent. Science.

[208] Bohrer L.R., Chen S., Hallstrom T.C., Huang H. (2010). Androgens suppress ezh2 expression via retinoblastoma (RB) and p130-dependent pathways: A potential mechanism of androgen-refractory progression of prostate cancer. Endocrinology.

[209] Hofman K., Swinnen J.V., Verhoeven G., Heyns W. (2001). E2f activity is biphasically regulated by androgens in lncap cells. Biochem. Biophys. Res. Commun..

[210] Cao P., Deng Z., Wan M., Huang W., Cramer S.D., Xu J., Lei M., Sui G. (2010). MicroRNA-101 negatively regulates EZH2 and its expression is modulated by androgen receptor and HIF-1α/HIF-1β. Mol. Cancer.

[211] Varambally S., Cao Q., Mani R.S., Shankar S., Wang X., Ateeq B., Laxman B., Cao X., Jing X., Ramnarayanan K. (2008). Genomic loss of microRNA-101 leads to overexpression of histone methyltransferase EZH2 in cancer. Science.

[212] Farber S., Diamond L.K. (1948). Temporary remissions in acute leukemia in children produced by folic acid antagonist, 4-aminopteroyl-glutamic acid. N. Engl. J. Med..

[213] Loening S.A., Beckley S., Brady M.F., Chu T.M., deKernion J.B., Dhabuwala C., Gaeta J.F., Gibbons R.P., McKiel C.F., McLeod D.G. (1983). Comparison of estramustine phosphate, methotrexate and cis-platinum in patients with advanced, hormone refractory prostate cancer. J. Urol..

[214] Saxman S., Ansari R., Drasga R., Miller M., Wheeler B., McClean J., Einhorn L. (1992). Phase III trial of cyclophosphamide versus cyclophosphamide, doxorubicin, and methotrexate in hormone-refractory prostatic cancer. A hoosier oncology group study. Cancer.

[215] Jones W.G., Fossa S.D., Verbaeys A.C., Droz J.P., Klijn J.G., Boven E., de Pauw M., Sylvester R. (1990). Low-dose fortnightly methotrexate in advanced prostate cancer. The eortc genito-urinary tract cancer cooperative group. Eur. J. Cancer.

[216] Linsalata M., Caruso M.G., Leo S., Guerra V., D’Attoma B., di Leo A. (2002). Prognostic value of tissue polyamine levels in human colorectal carcinoma. Anticancer Res..

[217] Canizares F., Salinas J., de las Heras M., Diaz J., Tovar I., Martinez P., Penafiel R. (1999). Prognostic value of ornithine decarboxylase and polyamines in human breast cancer: Correlation with clinicopathologic parameters. Clin. Cancer Res..

[218] Heston W.D. (1991). Prostatic polyamines and polyamine targeting as a new approach to therapy of prostatic cancer. Cancer Surv..

[219] Durie B.G., Salmon S.E., Russell D.H. (1977). Polyamines as markers of response and disease activity in cancer chemotherapy. Cancer Res..

[220] Kubota S., Okada M., Yoshimoto M., Murata N., Yamasaki Z., Wada T., Imahori K., Ohsawa N., Takaku F. (1985). Urinary polyamines as a tumor marker. Cancer Detect. Prev..

[221] Russell D.H. (1983). Clinical relevance of polyamines. Crit. Rev. Clin. Lab. Sci..

[222] Sakai S., Ito Y., Koide T., Tei K., Hara A., Sawada H. (1986). Detection of urinary polyamine by a new enzymatic differential assay. (III). Studies on urinary polyamines in patients with malignant genitourinary diseases. Hinyokika Kiyo.

[223] Chatel M., Darcel F., Quemener V., Hercouet H., Moulinoux J.P. (1987). Red blood cell polyamines as biochemical markers of supratentorial malignant gliomas. Anticancer Res..

[224] Cipolla B., Guille F., Moulinoux J.P., Bansard J.Y., Roth S., Staerman F., Corbel L., Quemener V., Lobel B. (1994). Erythrocyte polyamines and prognosis in stage D2 prostatic carcinoma patients. J. Urol..

[225] Cipolla B., Guille F., Moulinoux J.P., Quemener V., Staerman F., Corbel L., Lobel B. (1993). Polyamines and prostatic carcinoma: Clinical and therapeutic implications. Eur. Urol..

[226] Weiss T.S., Bernhardt G., Buschauer A., Thasler W.E., Dolgner D., Zirngibl H., Jauch K.W. (2002). Polyamine levels of human colorectal adenocarcinomas are correlated with tumor stage and grade. Int. J. Colorectal Dis..

[227] Bergeron C., Bansard J.Y., Le Moine P., Bouet F., Goasguen J.E., Moulinoux J.P., Le Gall E., Catros-Quemener V. (1997). Erythrocyte spermine levels: A prognostic parameter in childhood common acute lymphoblastic leukemia. Leukemia.

[228] Soda K. (2011). The mechanisms by which polyamines accelerate tumor spread. J. Exp. Clin. Cancer Res..

[229] Cipolla B., Moulinoux J.P., Quemener V., Havouis R., Martin L.A., Guille F., Lobel B. (1990). Erythrocyte polyamine levels in human prostatic carcinoma. J. Urol..

[230] Cheng L.L., Wu C.-L., Smith M.R., Gonzalez R.G. (2001). Non-destructive quantitation of spermine in human prostate tissue samples using HRMAS 1 H NMR spectroscopy at 9.4 T. FEBS Lett..

[231] McDunn J.E., Li Z., Adam K.P., Neri B.P., Wolfert R.L., Milburn M.V., Lotan Y., Wheeler T.M. (2013). Metabolomic signatures of aggressive prostate cancer. Prostate.

[232] Gupta S., Ahmad N., Marengo S.R., MacLennan G.T., Greenberg N.M., Mukhtar H. (2000). Chemoprevention of prostate carcinogenesis by α-difluoromethylornithine in tramp mice. Cancer Res..

[233] Kee K., Foster B.A., Merali S., Kramer D.L., Hensen M.L., Diegelman P., Kisiel N., Vujcic S., Mazurchuk R.V., Porter C.W. (2004). Activated polyamine catabolism depletes acetyl-coa pools and suppresses prostate tumor growth in tramp mice. J. Biol. Chem..

[234] Devens B.H., Weeks R.S., Burns M.R., Carlson C.L., Brawer M.K. (2000). Polyamine depletion therapy in prostate cancer. Prostate Cancer Prostatic Dis..

[235] Kadmon D. (1992). Chemoprevention in prostate cancer: The role of difluoromethylornithine (DFMO). J. Cell. Biochem. Suppl..

[236] Moulinoux J.P., Quemener V., Cipolla B., Guille F., Havouis R., Martin C., Lobel B., Seiler N. (1991). The growth of MAT-LyLu rat prostatic adenocarcinoma can be prevented in vivo by polyamine deprivation. J. Urol..

[237] Danzin C., Jung M.J., Grove J., Bey P. (1979). Effect of alpha-difluoromethylornithine, an enzyme-activated irreversible inhibitor of ornithine decarboxylase, on polyamine levels in rat tissues. Life Sci..

[238] Meyskens F.L., Simoneau A.R., Gerner E.W. (2014). Chemoprevention of prostate cancer with the polyamine synthesis inhibitor difluoromethylornithine. Recent Results Cancer Res..

[239] Simoneau A.R., Gerner E.W., Nagle R., Ziogas A., Fujikawa-Brooks S., Yerushalmi H., Ahlering T.E., Lieberman R., McLaren C.E., Anton-Culver H. (2008). The effect of difluoromethylornithine on decreasing prostate size and polyamines in men: Results of a year-long phase IIB randomized placebo-controlled chemoprevention trial. Cancer Epidemiol. Biomarkers Prev..

[240] Streiff R.R., Bender J.F. (2001). Phase I study of N1-N11-diethylnorspermine (DENSPM) administered TID for 6 days in patients with advanced malignancies. Investig. New Drugs.

[241] Wagner T., Jung M. (2012). New lysine methyltransferase drug targets in cancer. Nat. Biotechnol..

[242] Kaminskas E., Farrell A., Abraham S., Baird A., Hsieh L.S., Lee S.L., Leighton J.K., Patel H., Rahman A., Sridhara R. (2005). Approval summary: Azacitidine for treatment of myelodysplastic syndrome subtypes. Clin. Cancer Res..

[243] Fu S., Hu W., Iyer R., Kavanagh J.J., Coleman R.L., Levenback C.F., Sood A.K., Wolf J.K., Gershenson D.M., Markman M. (2011). Phase 1b–2a study to reverse platinum resistance through use of a hypomethylating agent, azacitidine, in patients with platinum-resistant or platinum-refractory epithelial ovarian cancer. Cancer.

[244] Iwata H., Sato H., Suzuki R., Yamada R., Ichinomiya S., Yanagihara M., Okabe H., Sekine Y., Yano T., Ueno K. (2011). A demethylating agent enhances chemosensitivity to vinblastine in a xenograft model of renal cell carcinoma. Int. J. Oncol..

[245] Kiziltepe T., Hideshima T., Catley L., Raje N., Yasui H., Shiraishi N., Okawa Y., Ikeda H., Vallet S., Pozzi S. (2007). 5-Azacytidine, a DNA methyltransferase inhibitor, induces ATR-mediated DNA double-strand break responses, apoptosis, and synergistic cytotoxicity with doxorubicin and bortezomib against multiple myeloma cells. Mol. Cancer Ther..

[246] Mao M., Tian F., Mariadason J.M., Tsao C.C., Lemos R., Dayyani F., Gopal Y.N., Jiang Z.Q., Wistuba II, Tang X.M. (2013). Resistance to braf inhibition in braf-mutant colon cancer can be overcome with pi3k inhibition or demethylating agents. Clin. Cancer Res..

[247] Ramachandran K., Gordian E., Singal R. (2011). 5-Azacytidine reverses drug resistance in bladder cancer cells. Anticancer Res..

[248] Singal R., Ramachandran K., Gordian E., Quintero C., Zhao W., Reis I.M. (2015). Phase I/II study of azacitidine, docetaxel, and prednisone in patients with metastatic castration-resistant prostate cancer previously treated with docetaxel-based therapy. Clin. Genitourin. Cancer.

[249] Yang L., Lin C., Jin C., Yang J.C., Tanasa B., Li W., Merkurjev D., Ohgi K.A., Meng D., Zhang J. (2013). Lncrna-dependent mechanisms of androgen-receptor-regulated gene activation programs. Nature.

[250] Wee Z.N., Li Z., Lee P.L., Lee S.T., Lim Y.P., Yu Q. (2014). EZH2-mediated inactivation of IFN-γ-JAK-STAT1 signaling is an effective therapeutic target in MYC-driven prostate cancer. Cell Rep..

[251] Beltran H., Prandi D., Mosquera J.M., Benelli M., Puca L., Cyrta J., Marotz C., Giannopoulou E., Chakravarthi B.V., Varambally S. (2016). Divergent clonal evolution of castration-resistant neuroendocrine prostate cancer. Nat. Med..

